# Supplementary material for: CDKN1A is a target for phagocytosis-mediated cellular immunotherapy in acute leukemia
Source: Nat Commun. 2022 Nov 8;13:6739. doi: 10.1038/s41467-022-34548-3 (PMC9643439; doi:10.1038/s41467-022-34548-3)
Supplement: Supplementary file 1 — Supplementary Information [file 41467_2022_34548_MOESM1_ESM.pdf]

Supplementary Information

CDKN1A is a target for phagocytosis-mediated cellular immunotherapy in acute leukemia

Awatef Allouch ***et al.***

Description of what is included in this file:

Supplementary Fig. 1 Characterization of macrophage phagocytosis of leukemia cells.

Supplementary Fig. 2 CD47 is a key determinant of the phagocytosis of leukemic T cells by macrophages.

Supplementary Fig. 3 Proinflammatory activation of phagocytic macrophages.

Supplementary Fig. 4 p21 knockdown does not modulate the cell cycle progression, proliferation or viability of macrophages.

Supplementary Fig. 5 p21 controls the phagocytosis of leukemic T cells.

Supplementary Fig. 6 CD47 blockade does not enhance the p21-mediated phagocytosis of MOLT4 cells.

Supplementary Fig. 7 p21 expression modulates SIRPα protein and membrane cell-surface expression.

Supplementary Fig. 8 Macrophages derived from p21-expressing genetically engineered monocytes do not exhibit changes in their cell cycle progression, viability, proliferation or proinflammatory activation status.

Supplementary Fig. 9 Establishment of the adoptive transfer protocol of human monocytes and a mouse model of human T-ALL.

Supplementary Fig. 10 Lentiviral transduction efficiency of MDMs derived from Co.TD-Mos or p21TD-Mos.

Supplementary Fig. 11 Biological parameters associated with p21TD-Mo-based cellular therapy for human T-ALL.

Supplementary Fig. 12 Persistence of p21-engineered macrophages in tumor-free mice.

Supplementary Fig. 13 p21TD-Mo-based therapy reduces the leukemic burden and prolongs survival in the human T-ALL model.

Supplementary Fig. 14 Clodronate-containing liposome-mediated depletion of $CFSE^+$ Co.TD-Mo- and p21TD-Mo-derived macrophages.

Supplementary Fig. 15 CD47 expression dictates the survival of $mCherry^+$ MOLT4 cell-engrafted mice.

Supplementary Fig. 16 p21TD-Mo-based therapy triggers proinflammatory reprogramming of TAMs in the human T-ALL model.

Supplementary Fig. 17 p21TD-Mo-based therapy does not trigger the pro-inflammatory activation of murine MDMs in the human T-ALL model.

Supplementary Fig. 18 Characterization of bone marrow invasion in mice engrafted with diagnosed or relapsed T-ALL-derived PDXs.

Supplementary Fig. 19 IFNγ reduces the viability of MOLT4 leukemia cells and prolongs the survival of $mCherry^+$ MOLT4 cell-engrafted NSG mice.

Supplementary Fig. 20 Representative sequential FACS sorting/gating strategies.

Supplementary Fig. 21 Representative sequential FACS gating strategies.

Supplementary Table 1 Characteristics of the AML patients providing the $CD34^+$ cells used in this study.

Supplementary Table 2 Characteristics of the T-ALL patients providing the PDX cells used in this study.

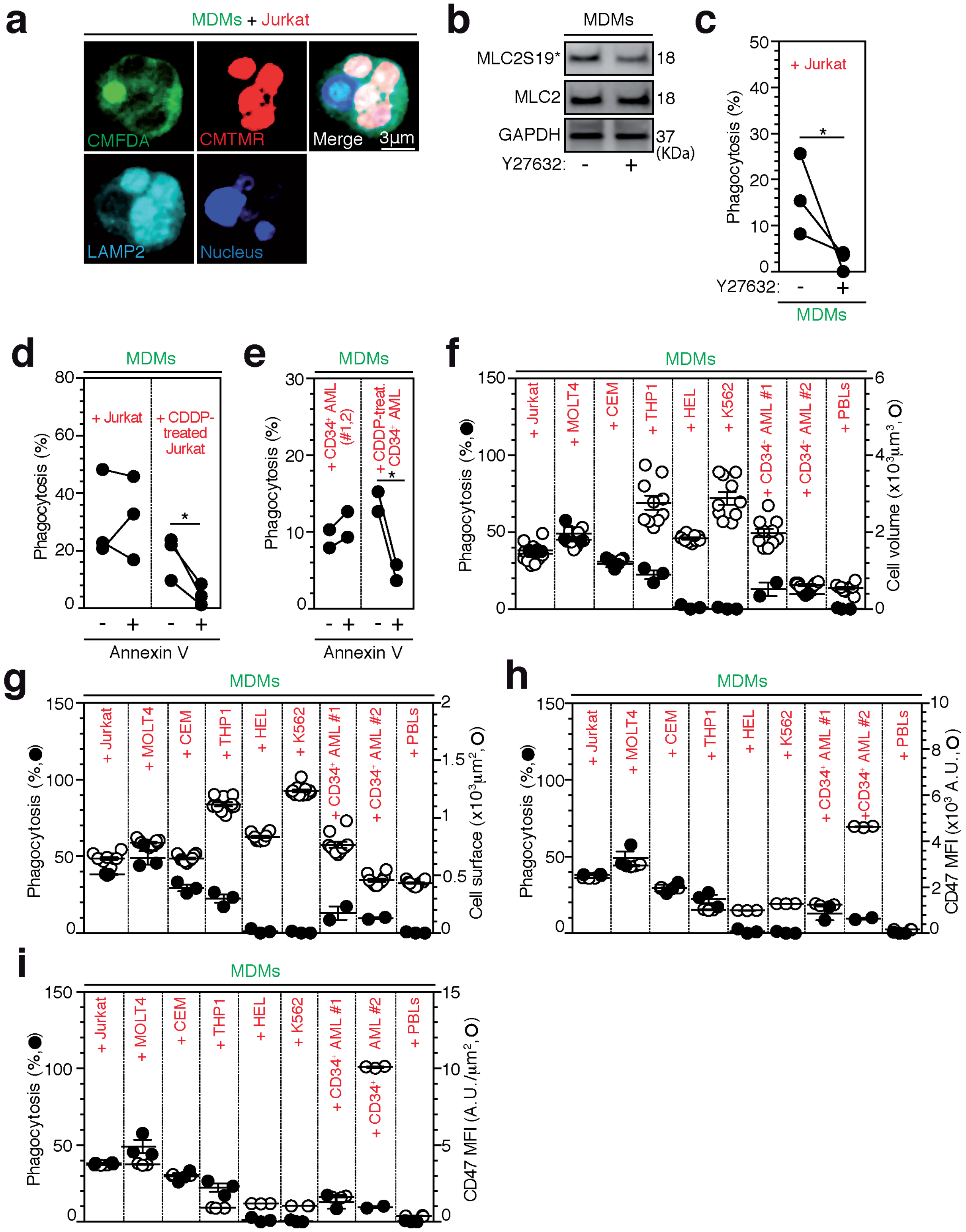

a
MDMs + Jurkat
CMFDA
CMTMR
Merge
3µm
LAMP2
Nucleus
b
MDMs
MLC2S19*
MLC2
GAPDH
18
18
37
(KDa)
Y27632: - +
c
+ Jurkat
Phagocytosis (%)
Y27632: - +
MDMs
d
MDMs
+ Jurkat
+ CDDP-treated Jurkat
Phagocytosis (%)
Annexin V
e
MDMs
+ CD34+ AML (#1,2)
+ CDDP-treat. CD34+ AML
Phagocytosis (%)
Annexin V
f
MDMs
+ Jurkat
+ MOLT4
+ CEM
+ THP1
+ HEL
+ K562
+ CD34+ AML #1
+ CD34+ AML #2
+ PBLs
Phagocytosis (%, ●)
Cell volume (x10³µm³, ○)
g
MDMs
Phagocytosis (%, ●)
Cell surface (x10³µm², ○)
h
MDMs
Phagocytosis (%, ●)
CD47 MFI (x10³ A.U., ○)
i
MDMs
Phagocytosis (%, ●)
CD47 MFI (A.U./µm², ○)

Supplementary Fig. 1 Characterization of macrophage phagocytosis of leukemia cells. a Confocal micrographs of CMFDA-labeled MDMs and CMTMR-labeled Jurkat cells cocultured for 8 h and stained for the lysosomal marker LAMP2. **b** Expression of Myosin Light Chain 2 (MLC2) and its phosphorylated/activated form (MLC2S19*) evaluated by WB analysis in MDMs treated for 24 h in the absence or presence of Y27632 (30 µM), a ROCK pathway inhibitor. **c** Percentages of phagocytosis of Jurkat cells by control- or Y27632-pretreated MDMs, as in (**b**), at 8 h of coculture in the presence or absence of Y27632 (30 µM) (*p=0.05). **d, e** Percentages of phagocytosis of viable or apoptotic (pretreated for 24 h with CDDP (50 µM)) Jurkat (*p=0.05) (**d**) or patient $CD34^+$ AML (*p=0.0331) (**e**) cells by MDMs in the presence or absence of human recombinant Annexin V (5 µg/ml) for 8 h of coculture. **f, g** Percentages of phagocytosis of the indicated leukemia cells or nontransformed PBLs by MDMs as a function of target cell volume (**f**) and surface (**g**), both determined after 3D reconstruction of 0.22-µm confocal microscopy z stacks by Volocity software. **h, i** Percentages of phagocytosis of the indicated leukemia cells or nontransformed PBLs by MDMs as a function of (**h**) membrane CD47 expression determined by FACS (**h**) or normalized (**i**) to the cell surface (**i**). In (**a, b**), the data are representative of n=3 donors. In (**c, d**) and (**e**), the data are donor matched from n=3 and n=2 donors. In (**f, g, h, i**), the data are presented as the mean±SEM from n=3 donors and for target cells n=10 (**f, g**) or n=3 (**h, i**) independent experiments. Exact p-values are indicated and determined with ordinary two-way ANOVA (**d, e**) and one-tailed Mann-Whitney (**c**) tests. Source data are provided as a Source Data file.

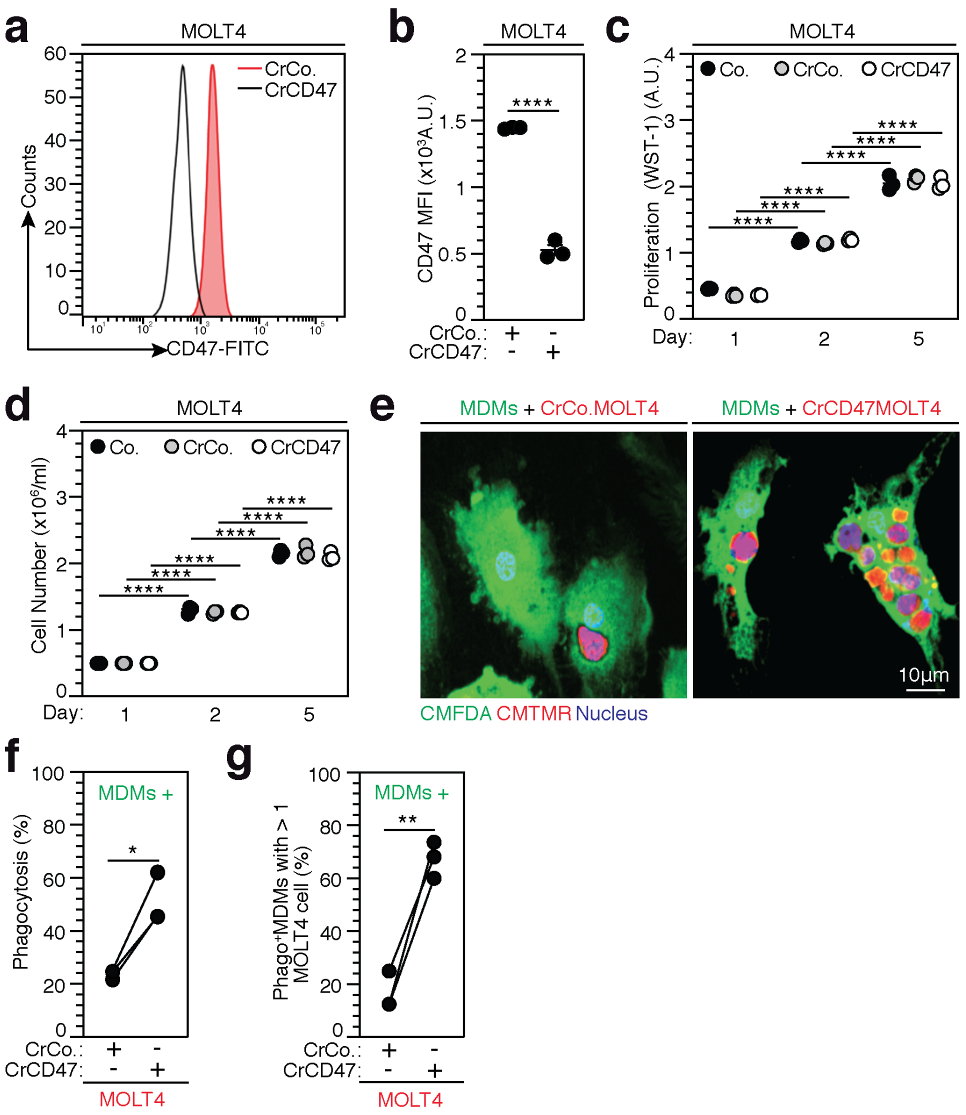

Supplementary Fig. 2 CD47 is a key determinant of the phagocytosis of leukemic T cells by macrophages. a, b FACS histograms (**a**) and cell-surface expression (MFI) (****p=2.1e-05) (**b**) of human CD47 (hCD47) on stable CD47-depleted (CrCD47MOLT4) or control (CrCo.MOLT4) MOLT4 cells obtained through transduction with lentiviral vectors encoding specific CD47 CRISPR guide RNAs (gRNAs) and the CAS9 gene or with control lentiviral vectors, respectively. **c**, **d**, Cell proliferation of CrCD47 and CrCo.MOLT4 cells assessed by a

WST-1 assay (****p=6.2e-11, ****p=1.5e-11, ****p=5.1e-12, ****p=2.7e-12, ****p=4.9e-13, ****p=4.1e-12) (**c**) and viable cell number determination (****p=1.1e-12, ****p=2.1e-12, ****p=2.1e-12, ****p=4.4e-13, ****p=1.8e-13, ****p=5.5e-13) (**d**) on d 1, 2 and 5. **e** Confocal micrographs of CMFDA-labeled MDMs and CMTMR-labeled CrCD47 or CrCo.MOLT4 cells co-cultured for 8 h. **f, g** Percentages of phagocytosis of CrCD47 or CrCo.MOLT4 cells by MDMs after 8 h of coculture (*p=0.0307) (**f**) and the related percentage of $Phago^{+}$ MDMs that engulfed more than one MOLT4 target cell (**p=0.0056) (**g**). (**a**) and (**e**) are data representative of n=3 independent experiments and n=3 donors. In (**b**, **c**, **d**), the data are presented as the mean±SEM from n=3 independent experiments. In (**f, g**), the data are donor matched from n=3 donors. Exact p-values are indicated and determined with two-tailed unpaired t (**b**), two-tailed (**f**) and one-tailed (**g**) paired t, and two-way ANOVA with Tukey's multiple comparison (**c**, **d**) tests. Source data are provided as a Source Data file.

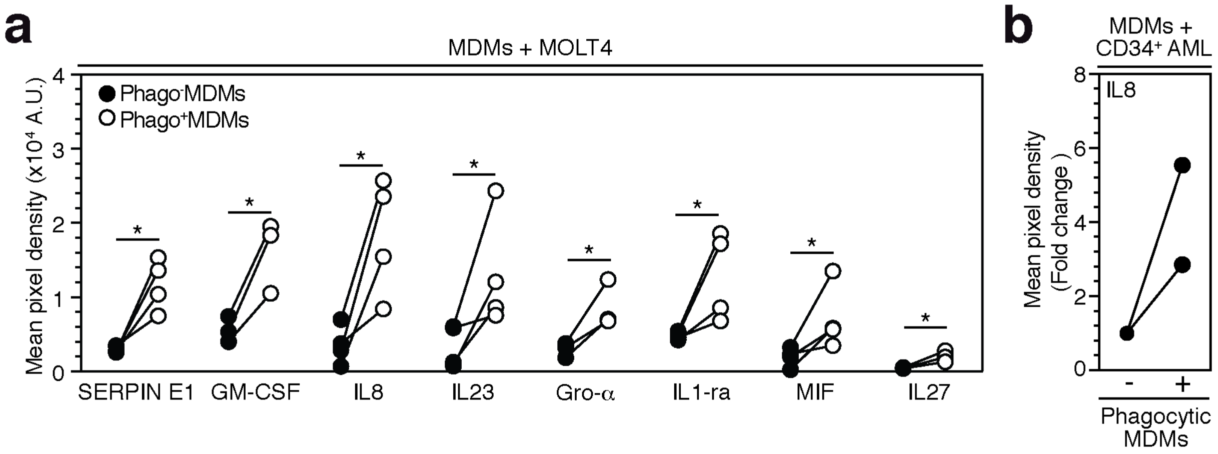

Supplementary Fig. 3 Proinflammatory activation of phagocytic macrophages. a Levels of secreted proinflammatory cytokines SERPIN E1, GM-CSF, IL8, IL23, Gro-α, IL1-ra, MIF and IL27 (*p=0.0143, *p=0.05, *p=0.0143, *p=0.0143, *p=0.05, *p=0.0143, *p=0.0143, *p=0.05, respectively) determined by a cytokine array at 96 h after sorting, in the cell supernatants of FACS-sorted Phago^{+} MDMs or Phago^{-} MDMs, as in **Fig. 1e**, which were obtained after 2 h of coculture of MDMs with MOLT4 cells. **b** Levels of secreted IL8, determined by a cytokine array at 96 h after sorting, in the cell supernatants of FACS-sorted Phago^{+} MDMs or Phago^{-} MDMs, as in **Fig. 1e**, which were obtained after 2 h of coculture of MDMs with CD34^{+} blasts. In (**a**) and (**b**), data are donor matched from (n=4 for SERPIN E1; n=3 for GM-CSF, Gro-α and IL27; n=4 for IL8, IL23, IL1-ra, MIF) and (n=2) donors. Exact p-values are indicated and determined with one-tailed unpaired Mann-Whitney (**a, b**) test. Source data are provided as a Source Data file.

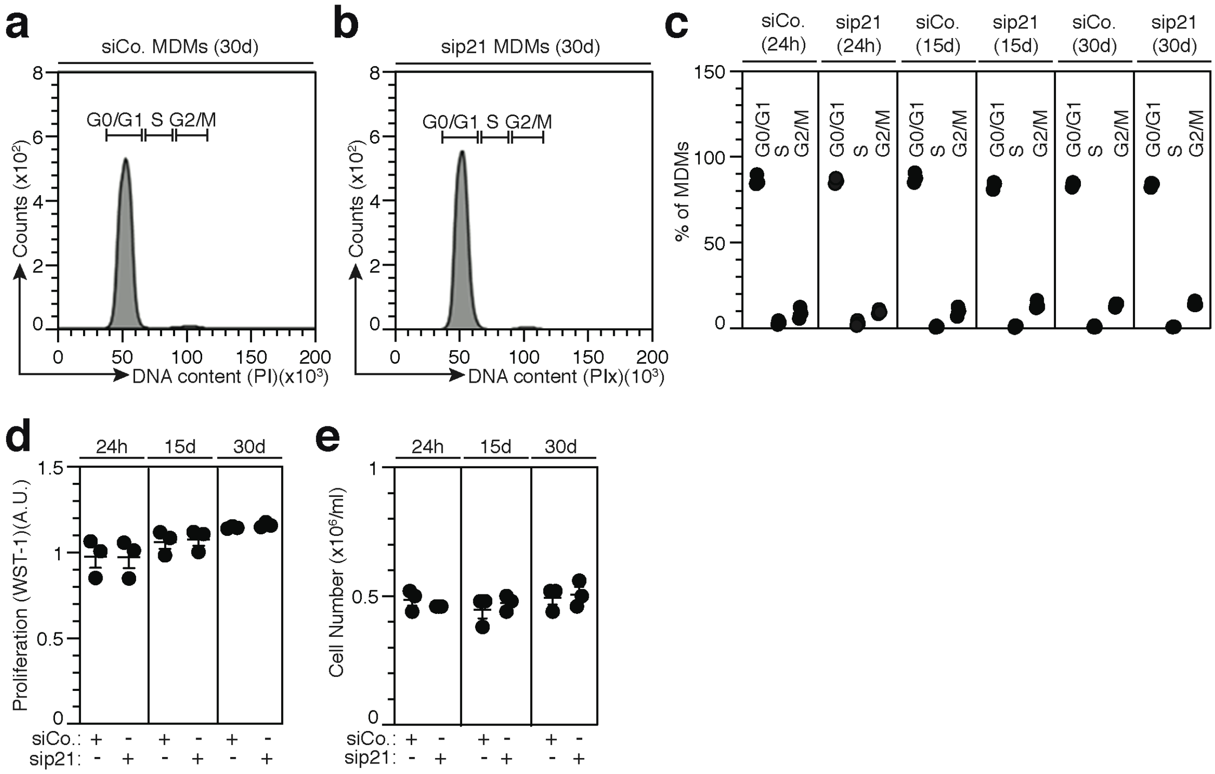

Supplementary Fig. 4 p21 knockdown does not modulate the cell cycle progression, proliferation or viability of macrophages. a-c FACS histograms (**a, b**) and percentages **(c)** of control (siCo.) or p21-knockdown (sip21) MDMs distributed in the G0/G1, S, G2/M phases of the cell cycle analyzed by DNA content staining with propidium iodide (PI) of, as shown in **Fig. 2a**, at 24 h, 15 d and 30 d after siRNA transfection. **d**, **e** Cell proliferation assessed by a WST-1 assay (**d**) and viability determined by measuring viable cell numbers (**e**) of siCo. or sip21 MDMs, as shown in **Fig. 2a**, at 24 h, 15 d and 30 d after siRNA transfection. In (**a**, **b**), data are representative of n=3 donors. In (**c, d, e**), the data are presented as the mean±SEM from n=3 donors. Source data are provided as a Source Data file.

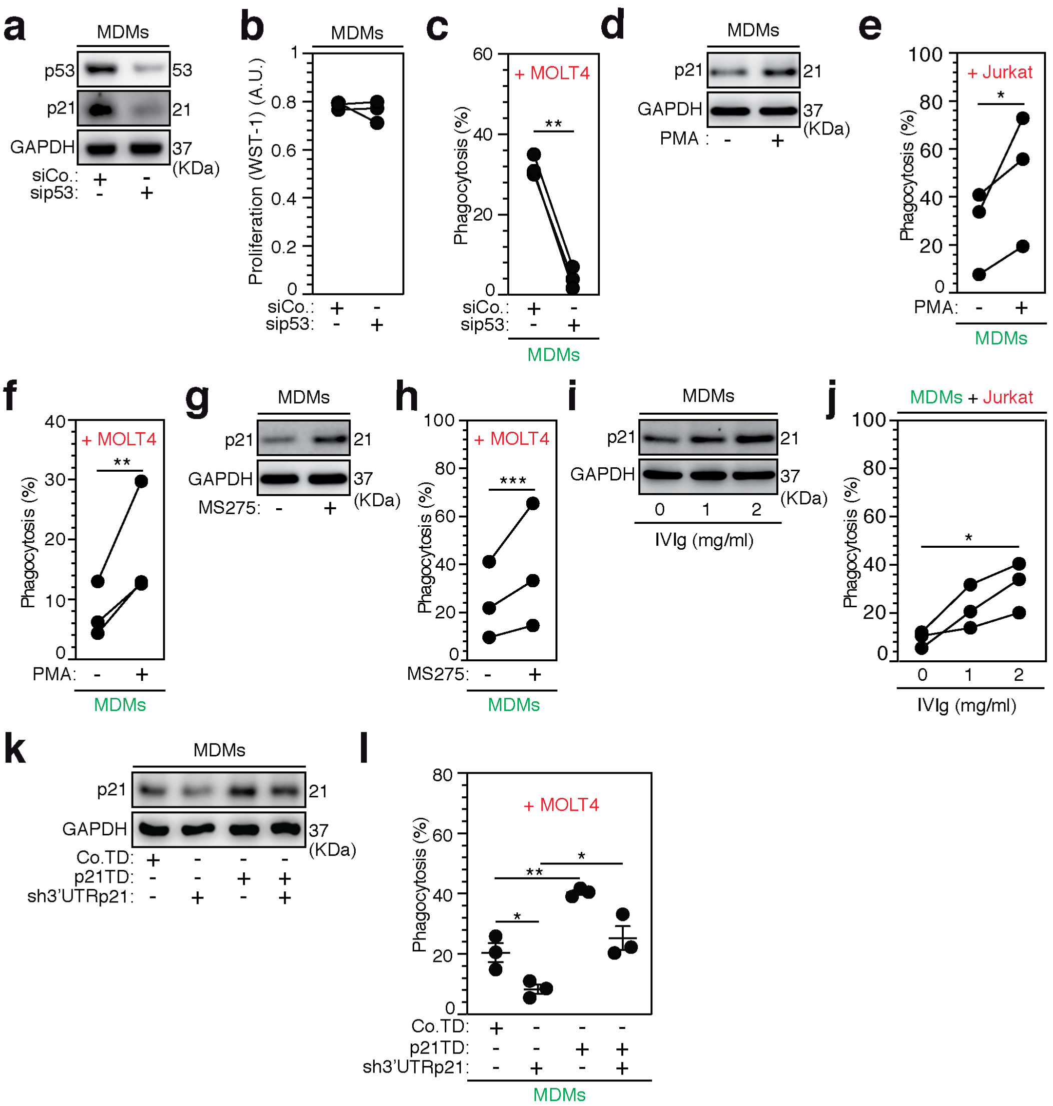

Supplementary Fig. 5 p21 controls the phagocytosis of leukemic T cells. a, b Expression of p53 and p21 determined by WB analysis (**a**) and cell proliferation assessed by a WST-1 assay (**b**) in control (siCo.) or p53-silenced (sip53) MDMs after 24 h siRNA transfection. **c**, Percentage of phagocytosis of MOLT4 cells by siCo. or sip53 MDMs detected after 8 h of coculture (**p=0.001). **d** Expression of p21 in control- or PMA (30 ng/ml)-treated MDMs determined by WB analysis at 32 h. **e**, **f** Percentage of phagocytosis of Jurkat (*p=0.0342) (**e**)

or MOLT4 (**p=0.0079) (**f**) cells by PMA-pretreated MDMs detected after 8 h of coculture. **g** Expression of p21 in control- or MS275 (1 μM)-treated MDMs determined by WB analysis at 32 h. **h** Percentage of phagocytosis of MOLT4 cells by MS275-pretreated MDMs detected after 8 h of coculture (***p=0.0007). **i** Expression of p21 by WB in control-treated or intravenous immunoglobulin (IVIg)-stimulated MDMs determined by WB analysis at 24h. **j** Percentage of phagocytosis of Jurkat cells by control-pretreated or IVIg-prestimulated MDMs detected after 8 h of coculture (*p=0.044). **k** Expression of p21 in MDMs transduced with control (Co.TD) lentiviral vectors, p21-encoding (p21TD) lentiviral vectors and/or lentiviral vectors encoding p21 short hairpin RNA targeting the 3' untranslated region of the p21 gene-encoding (sh3'UTRp21) at 72 h after transduction. **l** Percentage of phagocytosis of MOLT4 cells by the indicated transduced MDMs shown in (**k**) after 8 h of coculture (*p=0.0462, **p=0.045, *p=0.0103). In (**a**, **d**, **g**, **i**, **k**), the data are representative of n=3 donors. In (**b**, **c**, **e**, **f**, **h**, **j**), the data are donor matched from n=3 donors. The data in (**l**) are presented as the mean±SEM from n=3 donors. Exact p-values are indicated and determined with two-tailed paired t test (**c**), one-tailed ratio-paired t test (**e, f, h**) and one-way (**j**) or two-way (**l**) ANOVA with Tukey's multiple comparison tests. Source data are provided as a Source Data file.

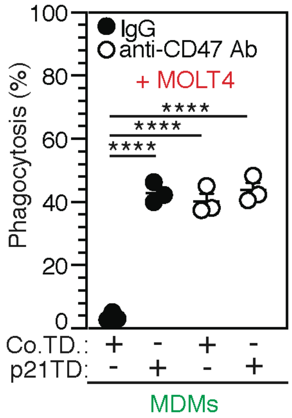

Supplementary Fig. 6 CD47 blockade does not enhance the p21-mediated phagocytosis of MOLT4 cells. The percentage of phagocytosis of MOLT4 cells by Co.TD-MDMs or p21TD-MDMs at 72 h after lentiviral transduction, as shown in **Fig. 2k**; the MDMs were precultured for 2 h in serum-free medium before the phagocytosis assay was performed with MOLT4 cells in the same medium for an additional 2 h in the presence of an isotype control (IgG) or anti-CD47 blocking antibody (B6H12.2 (7 µg/ml) (from down to up: ****p=3e-06; ****p=5e-06; ****p=3e-06). The data are presented as the mean±SEM from n=3 donors. Exact p-values are indicated and determined with two-way ANOVA with Tukey's multiple comparison test. Source data are provided as a Source Data file.

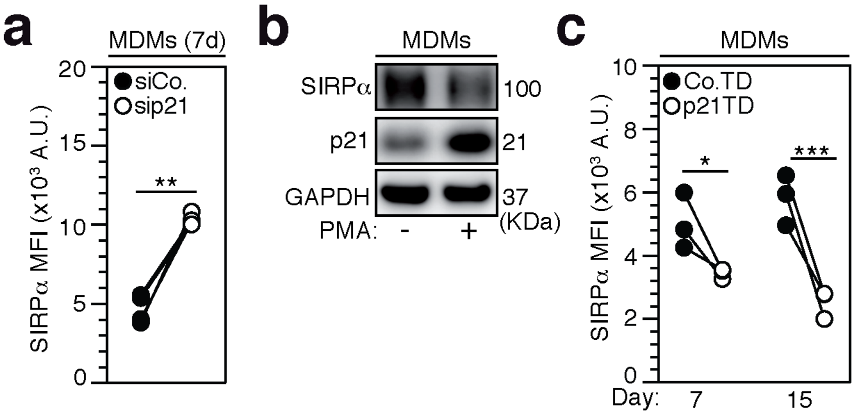

Supplementary Fig. 7 p21 expression modulates SIRPα protein and membrane cell surface expression. a SIRPα cell-surface expression of siCo. and sip21 MDMs, as shown in **Fig. 2h**, determined by FACS at 7 d after siRNA transfection (**p=0.0023). **b** Expression of SIRPα and p21 in control- or PMA-treated (30 ng/ml) MDMs determined by WB analysis at 32 h. **c** SIRPα cell-surface expression of Co.TD-MDMs or p21TD-MDMs, as shown in **Fig. 2k**, determined by FACS at 7 d and 15 d after lentiviral transduction (*p=0.0333,***p=0.0005). In (**b**), the data are representative of n=3 donors. In (**a**) and (**c**), the data are donor matched from n=4 and n=3 donors. Exact p-values are indicated and determined with a two-tailed paired t test (**a**) and two-way ANOVA with Sidak's multiple comparison (**c**) test. Source data are provided as a Source Data file.

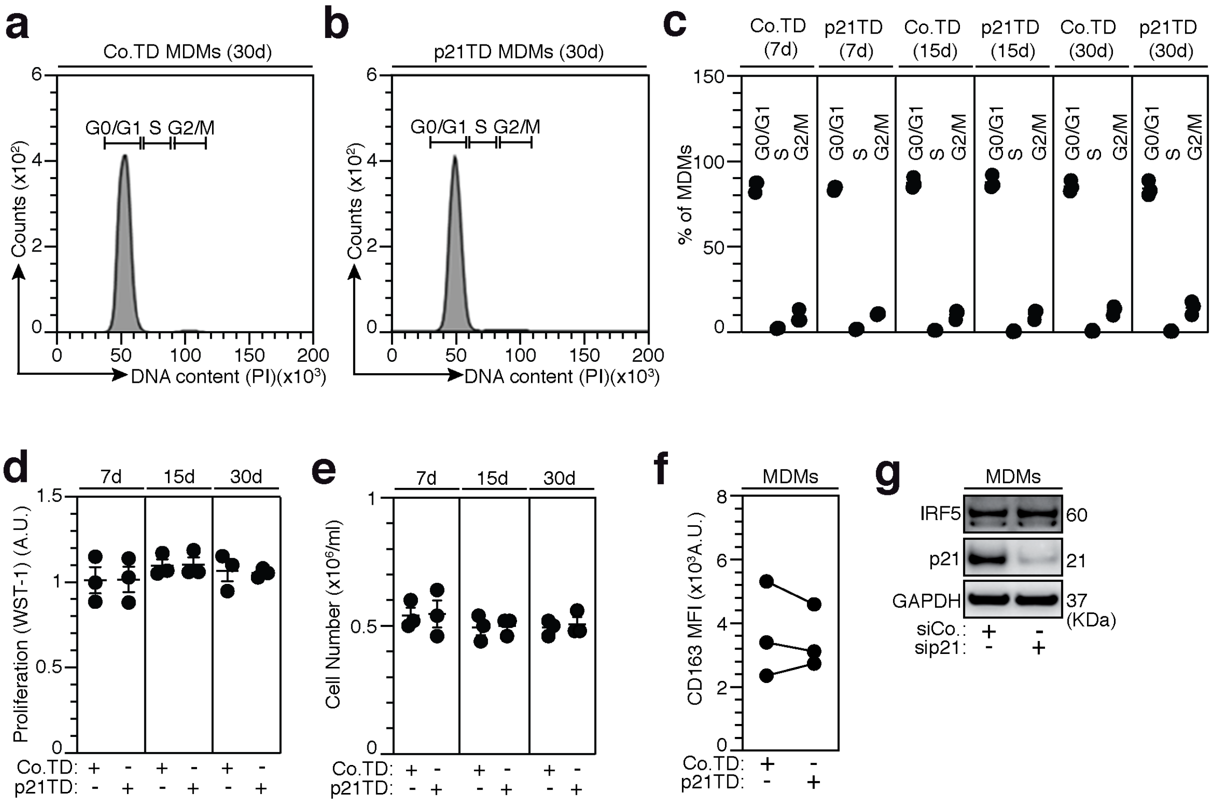

Supplementary Fig. 8 Macrophages derived from p21-expressing genetically engineered monocytes, do not exhibit changes in their cell cycle progression, viability, proliferation or pro-inflammatory activation status. a-c FACS histograms (**a, b**) and percentages (**c**) of MDMs derived from Co.TD or p21TD genetically engineered monocytes by differentiation for 7 d distributed in the G0/G1, S, G2/M phases of the cell cycle analyzed by DNA content staining with propidium iodide, as shown in **Fig. 2q**, at 7 d, 15 d and 30 d after lentiviral transduction. **d**, **e** Cell proliferation assessed by a WST-1 assay (**d**) and viability determined by measuring viable cell numbers (**e**) of Co.TD-MDMs or p21TD-MDMs, as shown in **Fig. 2q**, at 7 d, 15 d and 30 d after lentiviral transduction. **f** CD163 membrane expression (MFI) of Co.TD-MDMs or p21TD-MDMs, as shown in **Fig. 2q**, determined by FACS at 11 d after lentiviral transduction. **g** Expression of IRF5 and p21 in control-treated (siCo.) or p21-silenced (sip21) MDMs determined by WB analysis at 24 h after siRNA transfection. In (**a**, **b**,

g), the data are representative of n=3 donors. In (**c, d, e**), the data are presented as the mean±SEM from n=3 donors. In (**f**), the data are donor matched from n=3 donors. Source data are provided as a Source Data file.

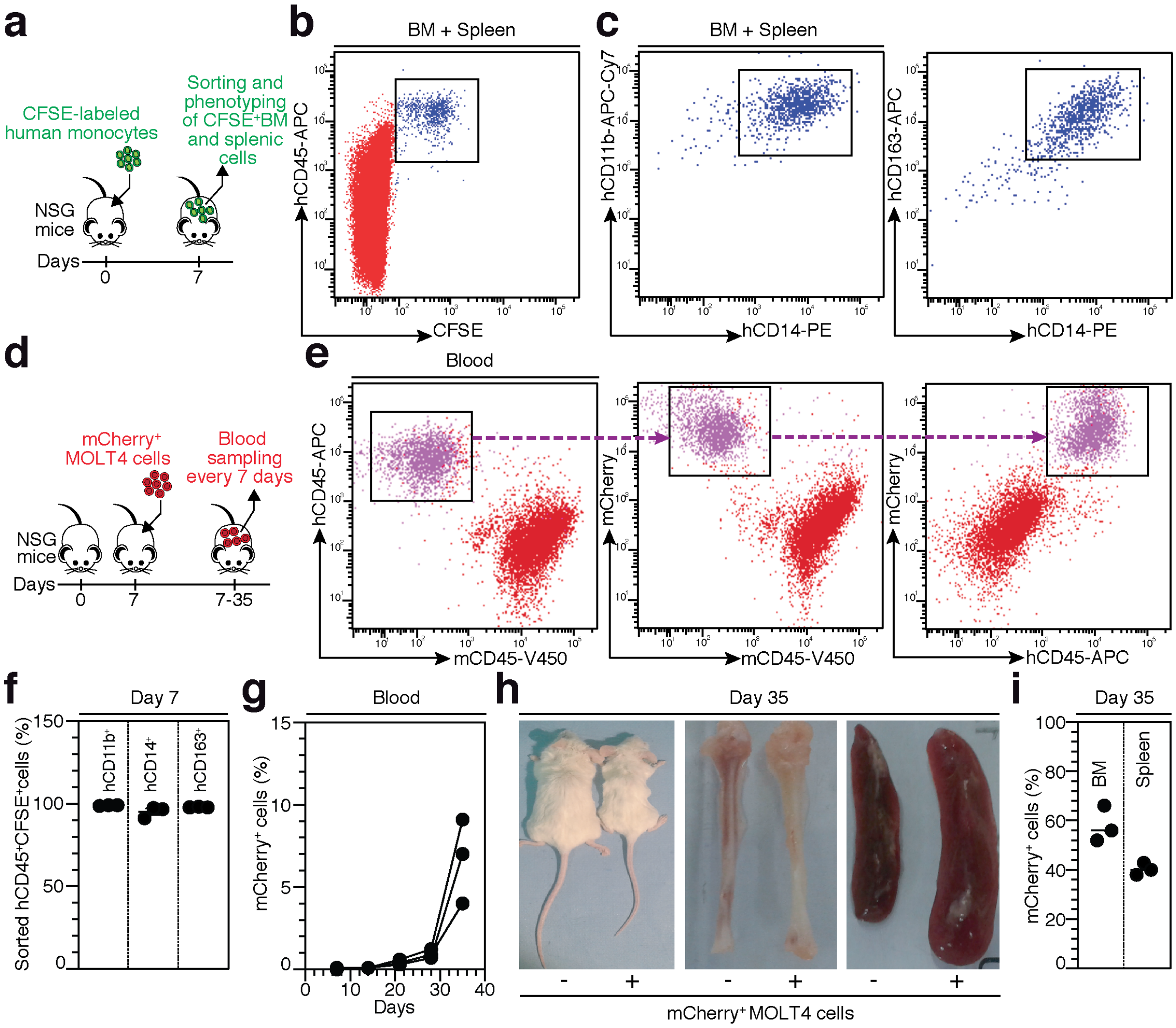

Supplementary Fig. 9 Establishment of the adoptive transfer protocol for human monocytes and a mouse model of human T-ALL. a Schematic representation of the protocol for the adoptive transfer of CFSE-labeled human monocytes via IV injections into NSG mice previously treated with total body irradiation (TBI), followed by sorting and immunophenotyping of CFSE$^+$ cells obtained from the bone marrow (BM) and spleen for human macrophage markers 7 d after monocyte transfer. **b, c** FACS dot plots of sorted CFSE$^+$ cells expressing the human leukocyte marker CD45 (hCD45) (**b**) in the BM and spleen of engrafted mice and the expression of the indicated human macrophage markers (**c**). **d** Schematic representation of the protocol for the engraftment of mCherry$^+$ MOLT4 cells into

NSG mice previously treated with TBI, including weekly peripheral blood sampling and FACS detection of leukemia cells. **e,** FACS dot plots showing the detection of mCherry$^+$ MOLT4 cells expressing hCD45, but not murine CD45 (mCD45)-expressing cells in the peripheral blood of engrafted mice at 35 d after leukemia cell injection. **f** Percentages of sorted hCD45$^+$CFSE$^+$ cells in (**b**) and (**c**) expressing the indicated macrophage markers . **g** Percentages of mCherry$^+$ MOLT4 cells detected at the indicated time points in the peripheral blood of mice engrafted as in (**d**). **h** Photographs of NSG mice engrafted (+) or not (-) with mCherry$^+$ MOLT4 cells, as in (**d**), and their corresponding BM and spleen 35 d after leukemia cell injection. **i,** Percentages of mCherry$^+$ MOLT4 cells in the BM and spleen of engrafted NSG mice. In (**b, c, e, h**), the data are representative of n=3 mice. In (**f, j**), the data are presented as the mean±SEM from n=3 mice. In (**g**), the data are mouse matched from n=3 mice. Source data are provided as a Source Data file.

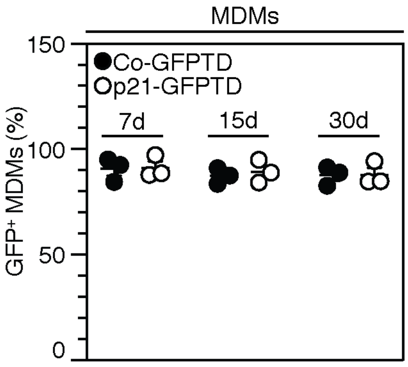

Supplementary Fig. 10 Lentiviral transduction efficiency of MDMs derived from Co.TD-Mos or p21TD-Mos. Percentage of live GFP-positive expressing MDMs (GFP^+ MDMs) derived *in vitro* from monocytes transduced with lentiviral vectors encoding p21 and GFP cDNAs (pp21-GFP) or a control vector (pCo-GFP), using the lentiviral transduction experimental procedure used for Co.TD-Mos and p21TD-Mos, and determined using immunofluorescence microscopy at d 7, 15 and 30 after transduction. The data are presented as the mean±SEM from n=3 donors. Source data are provided as a Source Data file.

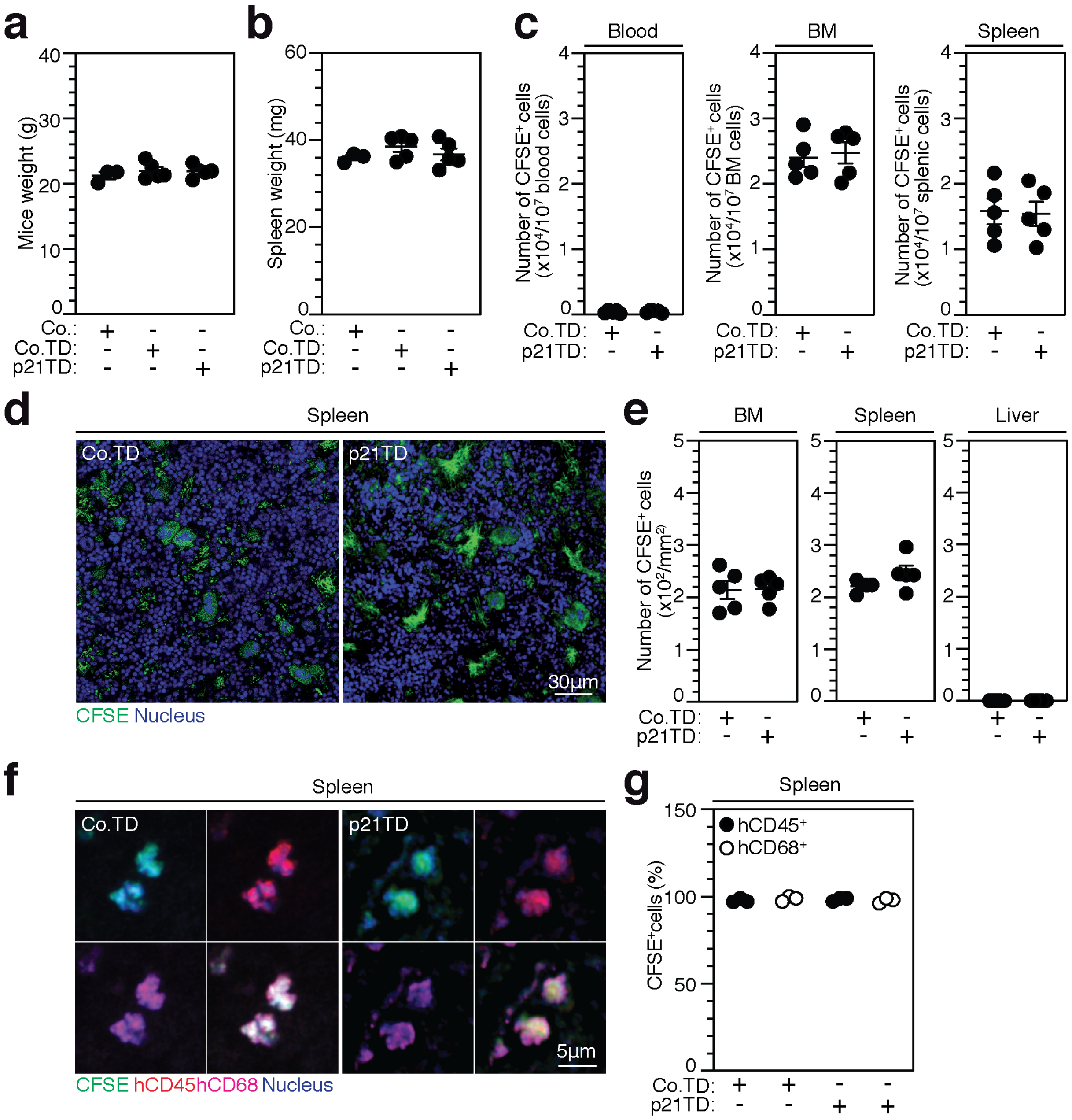

Supplementary Fig. 11 Biological parameters associated with p21TD-Mo-based therapy for human T-ALL. a, b Body (**a**) and spleen (**b**) weights of NSG mice infused with CFSE-labeled engineered monocytes (Mos) (Co.TD-Mos or p21TD-Mos) or controls (Co.). Weights were determined on d 21 after IV monocyte injection. **c** Numbers of CFSE$^+$ cells detected per 10^7 cells in the peripheral blood, bone marrow (BM) or spleen of mice (infused with CFSE-labeled Co.TD-Mos or p21TD-Mos) were determined by FACS at d 21 after Mo injection. **d**

Confocal micrographs showing CFSE^{+} cells in the spleen of mice infused with CFSE-labeled Co.TD-Mos or p21TD-Mos at 21 d after Mo transfer. **e** Numbers of CFSE^{+} cells in 1 mm^{2} of BM, spleen or liver tissue obtained from mice infused with CFSE-labeled Co.TD-Mos or p21TD-Mos and imaged by confocal microscopy 21 d after Mo injection. **f, g** Confocal micrographs (**f**) and percentage (**g**) of CFSE^{+} cells detected in mouse spleens evaluated 21 d after Mo transfer and expressing human leukocyte (hCD45) and human macrophage (hCD68) markers. In (**d**) and (**f**), the data are representative of n=5 and n=3 mice/group. In (**a, b**), the data are presented as the mean±SEM from n=3 mice for the Co. group and n=5 mice for the Co.TD and p21TD groups. In (**c**, **e**) and (**g**), the data are presented as the mean±SEM from n=5 and n=3 mice/group. Source data are provided as a Source Data file.

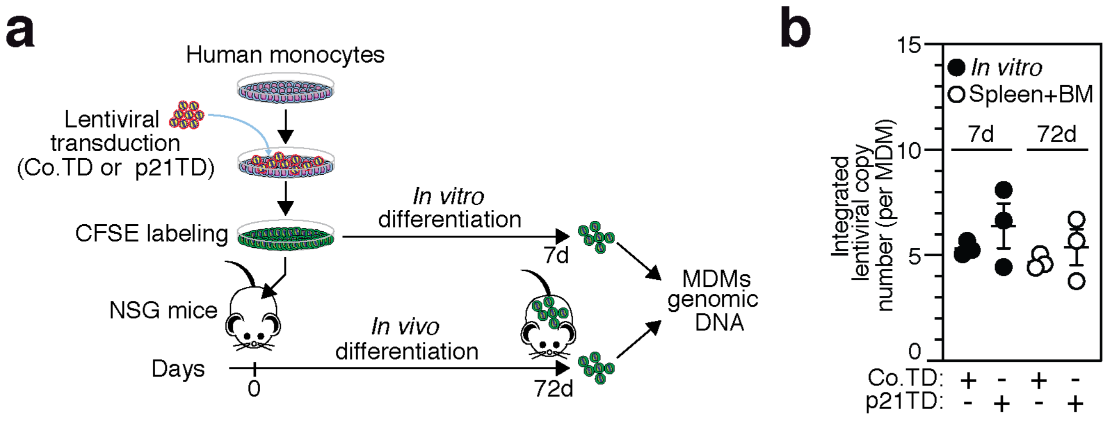

Supplementary Fig. 12 Persistence of p21-engineered macrophages in tumor-free mice. a, b Schematic representation showing the experimental procedure used to analyze the persistence of p21-engineered macrophages (**a**), and quantification of integrated lentiviral copy numbers per MDM (**b**) among MDMs derived from Co.TD-Mos or p21TD-Mos that were either differentiated *in vitro* for 7 d or sorted (hCD45^{+}hCD11b^{+}hCD71^{+}hCD14^{+}) from the BM and spleen on d 72 after adoptive transfer into tumor-free NSG mice. Integrated lentiviral copy numbers were determined from genomic DNA analyzed by qPCR. In (**b**), the data are presented as the mean±SEM from n=3 donors (*in vitro*) and n=3 mice/group (*in vivo*). Source data are provided as a Source Data file.

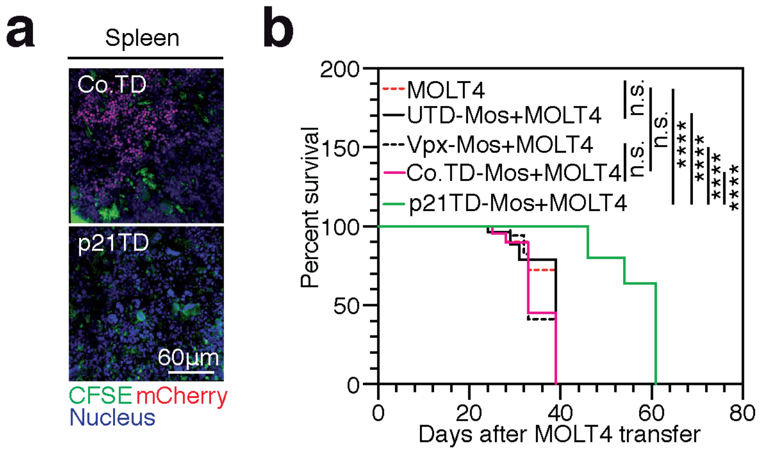

Supplementary Fig. 13 p21TD-Mo-based therapy reduces the leukemic burden and prolongs survival in the human T-ALL model. a Confocal micrographs showing mCherry$^+$ MOLT4 cells and CFSE$^+$ cells in the spleen of engrafted NSG mice 21 d after Mo transfer, as shown in **Fig. 3a**. **b** Survival of mCherry$^+$ MOLT4 cell-engrafted NSG mice not treated with adoptive transfer or adoptively transferred, as in **Fig. 3a**, with untransduced Mos (UTD-Mos), Vpx viral-like particle-treated Mos (Vpx-Mos), Co.TD-Mos or p21TD-Mos (from up to down: p=0.8831 (n.s.), p=0.8632 (n.s.), p=0.5617 (n.s.), ****p=1.1e-05, ****p=1.1e-05, ****p=2.2e-06, ****p=4.6e-06). In (**a**), the data are representative of n=5 mice/group. In (**b**), the survival data represent n=5 mice/group. Exact p-values are indicated and determined with the log-rank Mantel-Cox (**b**) test. Source data are provided as a Source Data file.

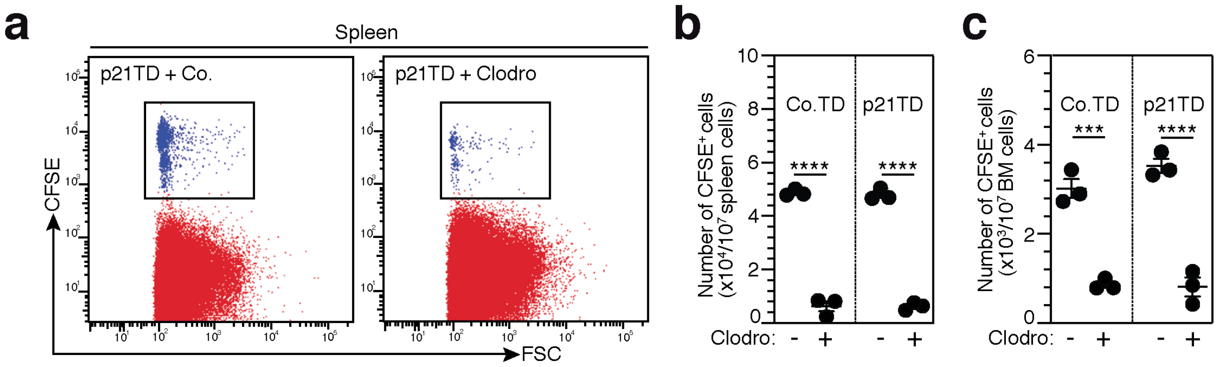

Supplementary Fig. 14 Clodronate-containing liposome-mediated depletion of CFSE$^+$ Co.TD-Mo- and p21TD-Mo-derived macrophages. a FACS dot plots of CFSE$^+$ spleen cells obtained from p21TD-Mo-transferred NSG mice, as in **Fig. 3a**, which were treated with control (Co.) or clodronate (Clodro)-containing liposomes 21 d after Mo transfer and assessed for specific depletion by FACS at 24 h after treatment. **b, c** Numbers of CFSE$^+$ cells detected per 10^7 cells from the spleen (****p=4.1e-08, ****p=4.7e-08) (**b**) or bone marrow (BM) (***p=0.0001, ****p=1.8e-05) (**c**) obtained from mice that received Co.TD-Mos or p21TD-Mos, as shown in **Fig. 3a**, and were treated with Co. or Clodro-containing liposomes 21 d after Mo transfer. The depletion of CFSE$^+$ cells at 24 d after liposomal treatment was evaluated by FACS. In (**a**), the data are representative of n=3 mice/group. In (**b, c**), the data are presented as the mean±SEM from n=3 mice/group. Exact p-values are indicated and determined with one-way ANOVA with Tukey's multiple comparison (**b, c**) test. Source data are provided as a Source Data file.

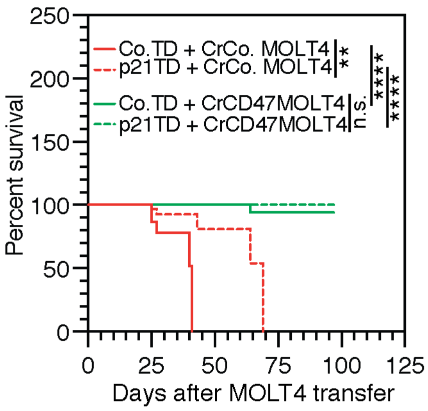

Supplementary Fig. 15 CD47 expression dictates the survival of mCherry^{+} MOLT4 cell-engrafted mice. Survival of NSG mice engrafted with stable CD47-depleted (CrCD47MOLT4) or control (CrCo.MOLT4) MOLT4 cells that received Co.TD-Mos or p21TD-Mos (from left to right: **p=0.0054, p=0.2781 (n.s.), ****p=7.8e-09, ****p=6.1e-06). The survival data are from n=5 mice/group. Exact p-values are indicated and determined with the log-rank Mantel-Cox test.

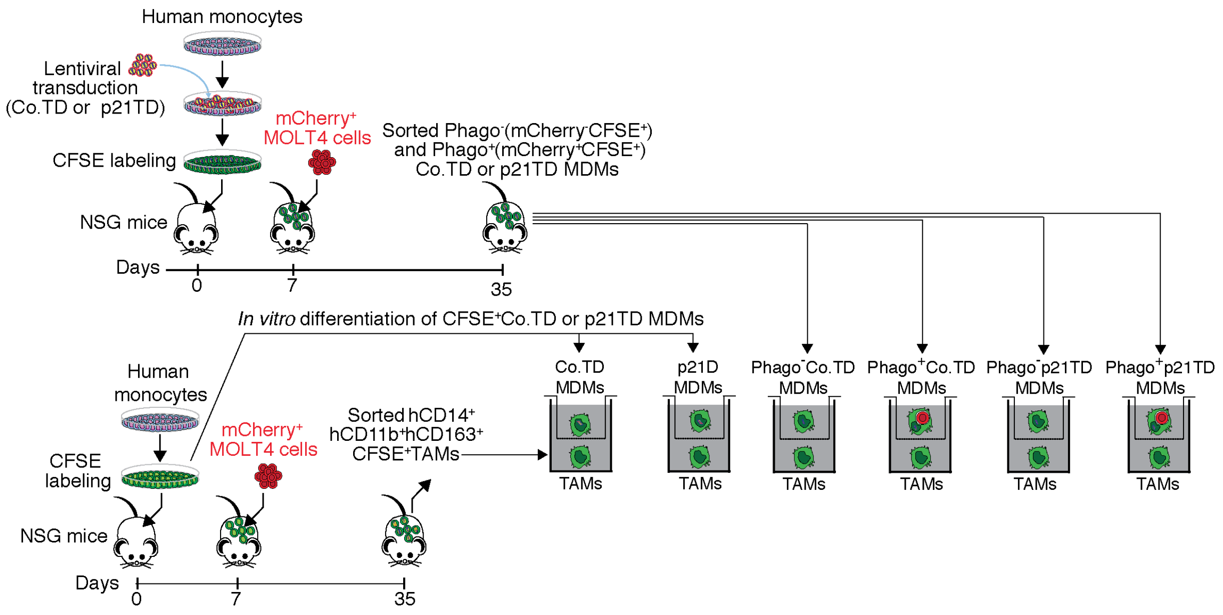

Supplementary Fig. 16 p21TD-Mo-based therapy triggers proinflammatory reprogramming of TAMs in the human T-ALL model. Schematic representation showing the experimental procedure used to determine the percentage of iNOS^{+} TAMs, using immunofluorescence microscopy (data shown in **Fig. 4f**). hCD14^{+}hCD11b^{+}hCD163^{+} CFSE^{+} MDMs (TAMs) were sorted from the BM and spleen of mCherry^{+} MOLT4 cell-engrafted NSG mice by FACS at day 35 after human Mo transfer, and cocultured in the bottom chamber of Transwell devices with Phago^{-} MDMs or Phago^{+} MDMs (in the upper chambers), which were sorted from the BM and the spleen of mCherry^{+} MOLT4 cell-engrafted NSG mice adoptively transferred with Co.TD-Mos or p21TD-Mos (cells collected at 35 d after Mo transfer). Control TAMs were cocultured with Co.TD-MDMs or p21TD-MDMs obtained by *in vitro* differentiation of Co.TD-Mos or p21TD-Mos. After 15 d of coculture, the percentage of iNOS^{+} TAMs was determined by immunofluorescence staining and microscopy. Source data are provided as a Source Data file.

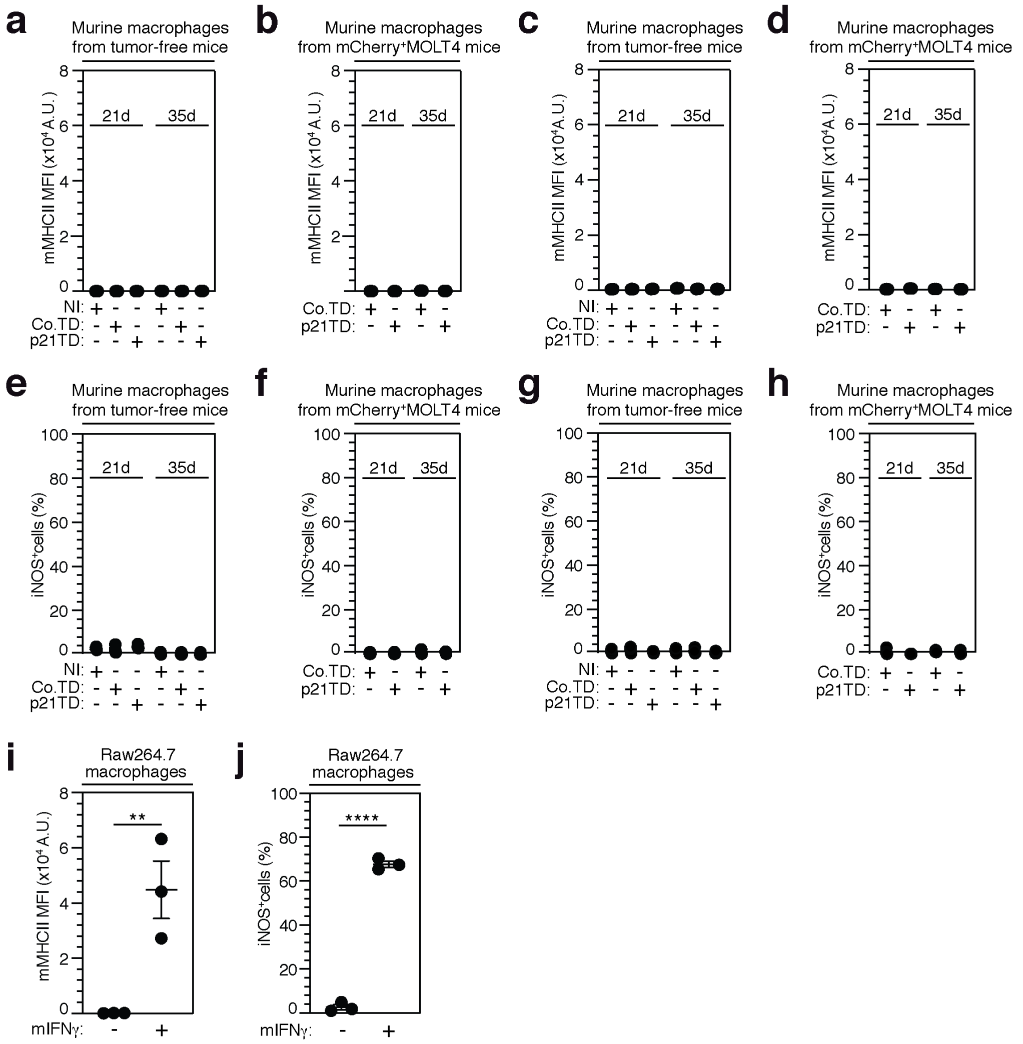

Supplementary Fig. 17 p21TD-Mo-based therapy does not trigger the proinflammatory activation of murine macrophages in the human T-ALL model. a-j Cell-surface expression (MFI) of murine MHCII (mMHCII) and the percentage of cells expressing iNOS ($iNOS^+$) were analyzed by FACS (**a-d**) and immunofluorescence staining and microscopy (**e-h**) of murine macrophages ($mCD45^+mF4/80^+$). Murine macrophages were sorted from the BM (**a, b, e, f**) and the spleen (**c, d, g, h**) of tumor-free (**a, c, e, g**) or $mCherry^+$ MOLT4 cell-engrafted (**b, d, f, h**) NSG mice on d 21 and d 35 after adoptive transfer of Co.TD-Mos or p21TD-Mos or harvested from mice not treated with infusion (NI). Proinflammatory activated murine Raw264.7 macrophages treated with murine IFNγ (mIFNγ) (1 μg/ml) for 48 h were

used as positive controls for mMHCII (**p=0.0063) (**i**) and iNOS (****p=3.6e-05) (**j**) staining. In (**a-h**) and (**i, j**), the data are presented as the mean±SEM from n=3 mice/group and n=3 independent experiments. Exact p-values are indicated and determined with one-tailed (**i**) and two-tailed (**j**) unpaired t tests. Source data are provided as a Source Data file.

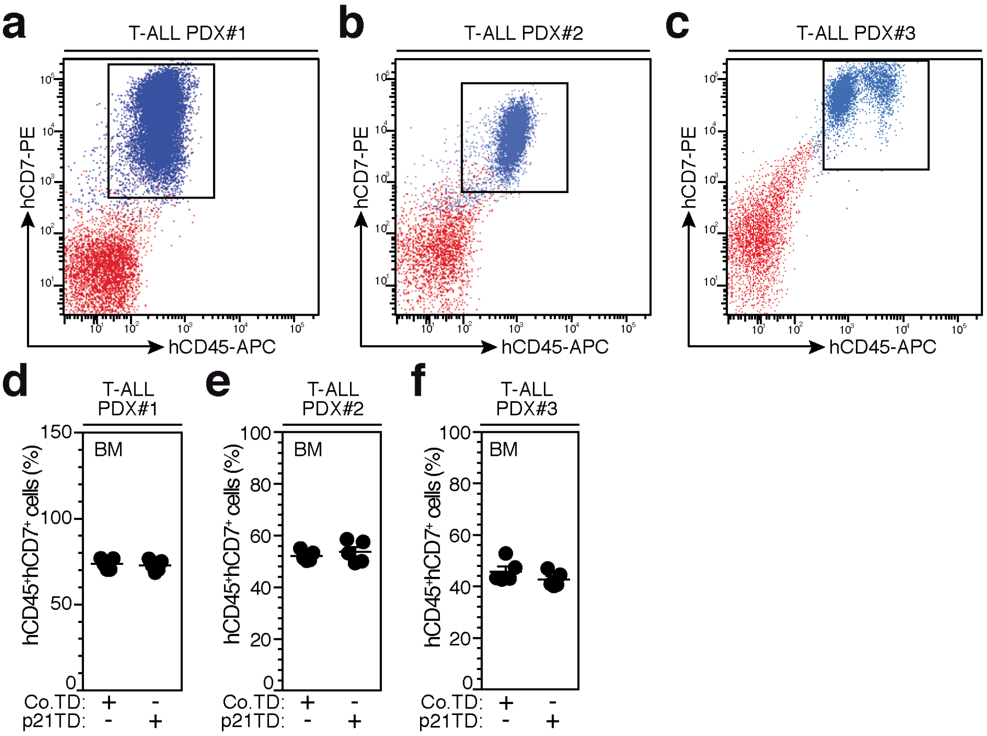

Supplementary Fig. 18 Characterization of bone marrow invasion in mice engrafted with diagnosed or relapsed T-ALL-derived PDXs. a-c FACS dot plots showing the bone marrow invasion in mice engrafted with T-ALL PDX#1 (**a**), PDX#2 (**b**) or PDX#3 (**c**), as shown in **Fig. 5a**. PDX#1 and PDX#2 were from the same patient but isolated at the diagnosis and relapse stages, respectively. Bone marrow (BM) invasion was assessed by FACS detection of human leukocyte (hCD45) and T-ALL (hCD7) markers 24 h before adoptive transfer of Co.TD-Mos or p21TD-Mos. **d-f** The percentages of $hCD45^{+}hCD7^{+}$ cells detected in the BM of mice engrafted with T-ALL PDX#1 (**d**), PDX#2 (**e**) or PDX#3 (**f**), as shown in **Fig. 5a**, were determined by FACS 24 h before adoptive transfer of Co.TD-Mos or p21TD-Mos. In (**a, b, c**), the data are representative of n=10 mice. In (**d, e, f**), the data are presented as the mean±SEM from n=5 mice/group. Source data are provided as a Source Data file.

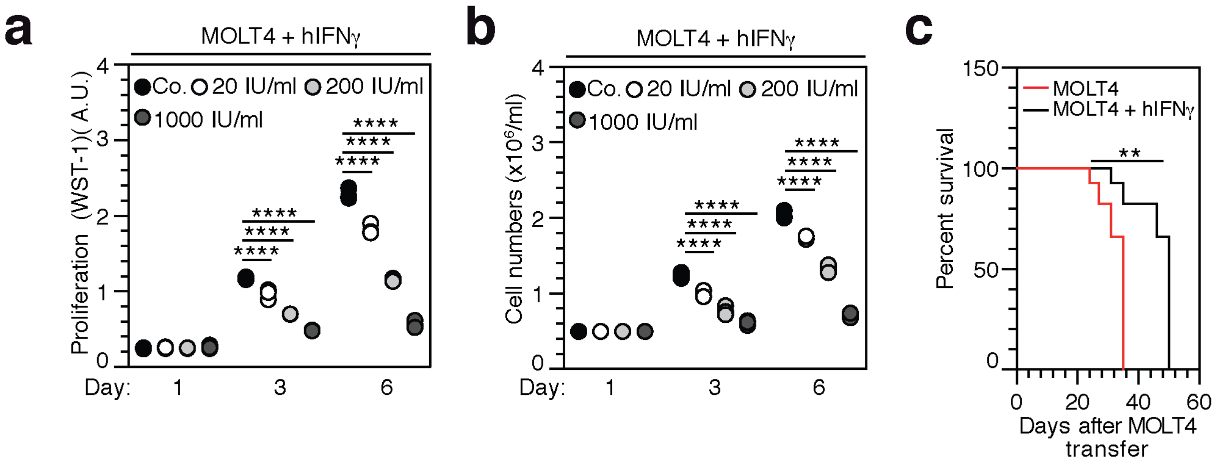

Supplementary Fig. 19 IFNγ reduces the viability of MOLT4 leukemia cells and prolongs the survival of mCherry^{+} MOLT4 cell-engrafted NSG mice. a, b Cell proliferation (using a WST-1 assay (from left to right: ****p=7.5e-05, ****p=9e-12, ****p=1.9e-14, ****p=1.2e-11, ****p=1.8e-14 and ****p=1.8e-14)) (**a**) and viability (viable cell counts) (from left to right: ****p=3.8e-07, ****p=1.5e-12, ****p=1.9e-14, ****p=1e-08, ****p=1.8e-14 and ****p=1.8e-14)) (**b**) of MOLT4 cells treated (or not) with the indicated hIFNγ concentrations (in International Units/ml (IU/ml)) and during the indicated times are shown. **c** Survival of mCherry^{+} MOLT4 cell-engrafted NSG mice treated (or not) with hIFNγ (10 μg/mouse) on d 7 after leukemia cell engraftment (**p=0.0028). In (**a, b**), the data represent the mean±SEM from n=3 independent experiments. In (**c**), the survival data are from n=5 mice/group. Exact p-values are indicated and determined with two-way ANOVA with Tukey's test (**a**, **b**) and the log-rank Mantel-Cox (**c**) test. Source data are provided as a Source Data file.

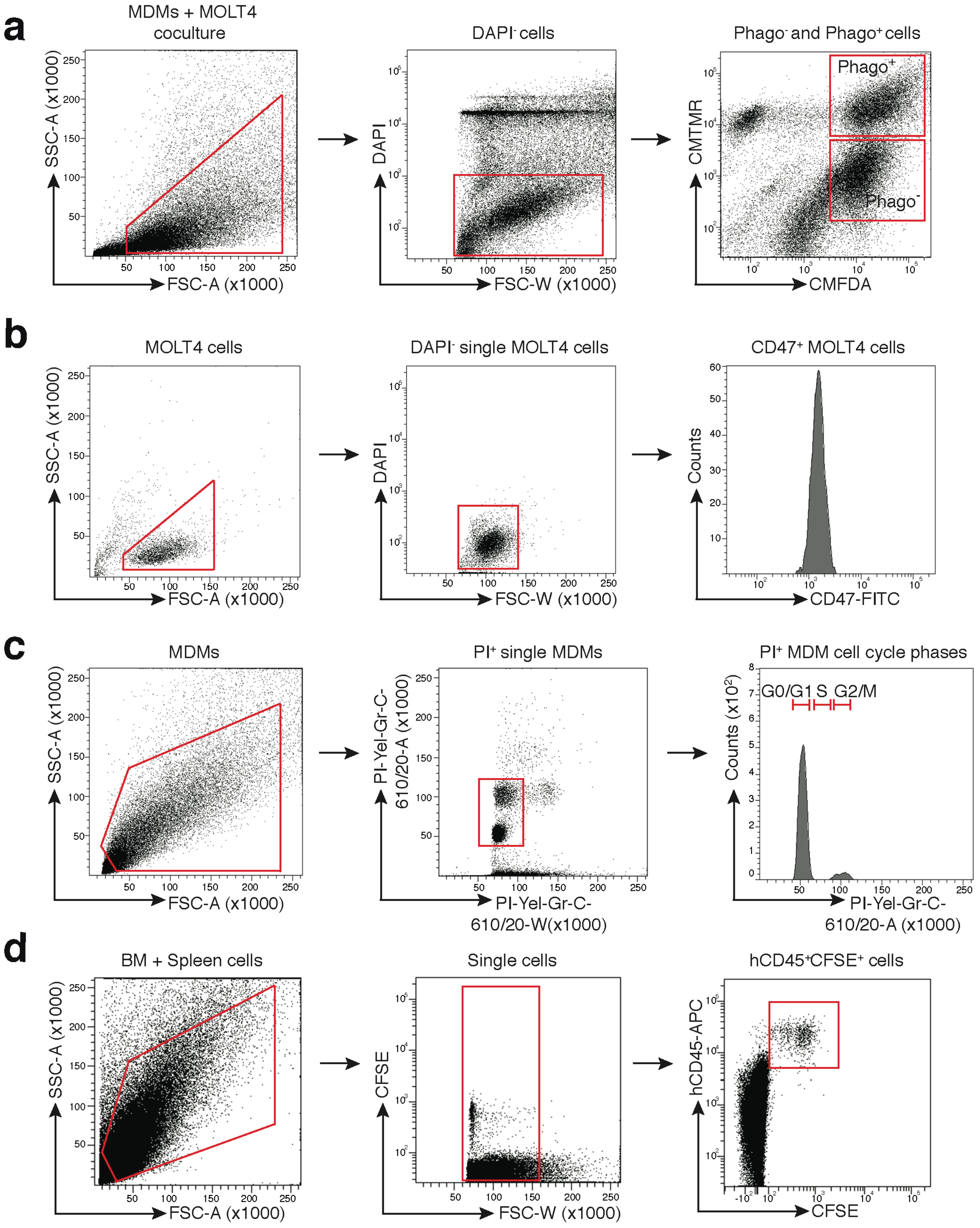

Supplementary Fig. 20. Representative sequential FACS sorting/gating strategies. a Representative FACS sorting strategy for **Fig. 1 e**. **b** Representative FACS gating strategy for **Supplementary Fig. 2a**. **c** Representative FACS gating strategy for **Supplementary Fig. 4a**,

b and **Supplementary Fig. 8a**, **b**. **d** Representative FACS sorting strategy for **Supplementary Fig. 9b**.

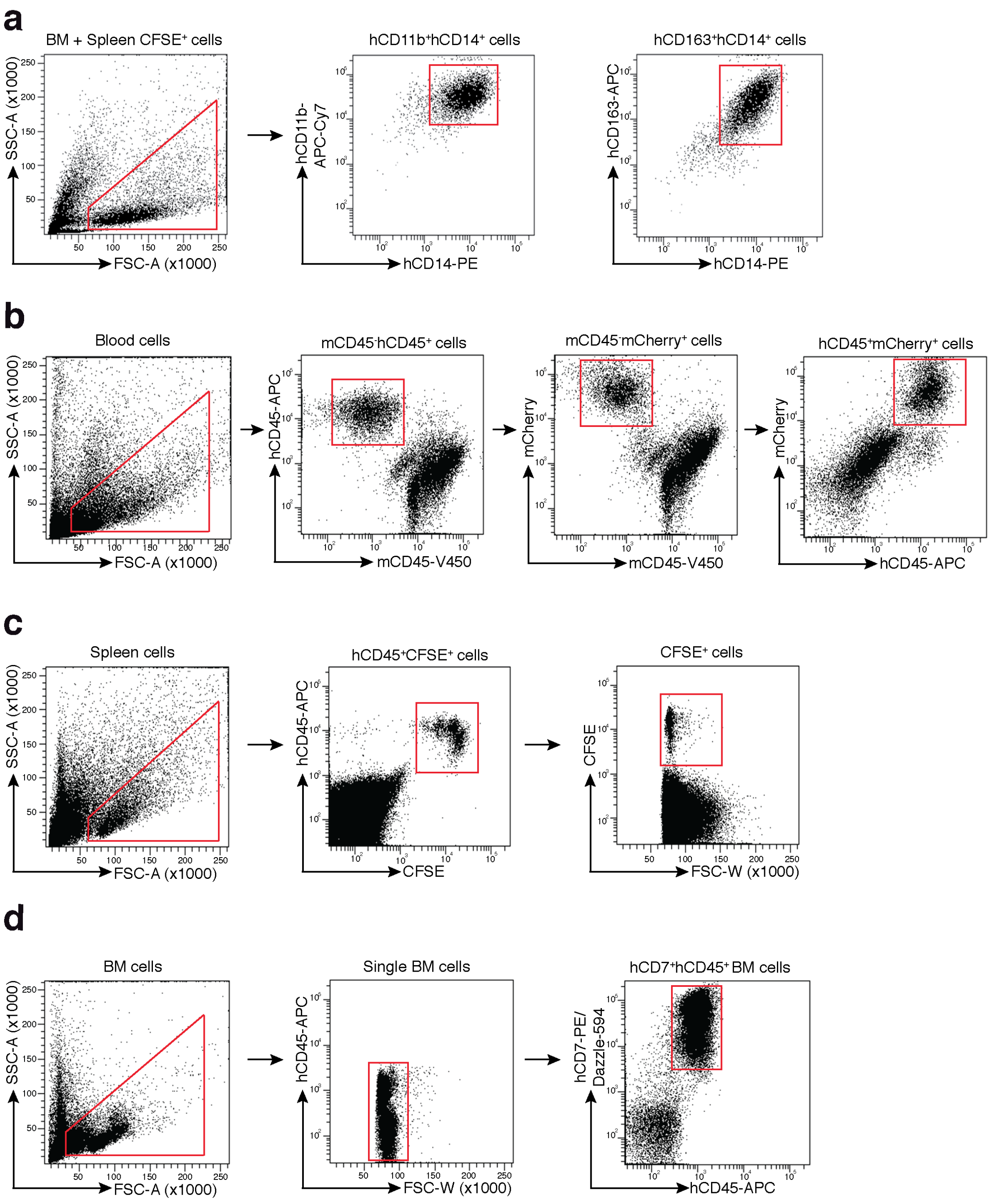

Supplementary Fig. 21. Representative sequential FACS gating strategies. a Representative FACS gating strategy for **Supplementary Fig. 9c**. **b** Representative FACS

gating strategy for **Supplementary Fig. 9e**. **c** Representative FACS gating strategy for **Supplementary Fig. 14a**. **d** Representative FACS gating for **Supplementary Fig. 18a**, **b, c**.

Supplementary Table 1 Characteristics of AML patients providing the CD34$^+$ cells used in this study.

	Age at sampling time (years)	Sex	WHO diagnosis	WHO diagnosis before AML	Sampled organs	WBC (10^9/L)	WBC monocytes (10^9/L)	Blasts in BM (%)	Karyotypes	Additional gene mutations	Multi-agent received treatments
1	73	Men	AML	CMML	PB	17.70	5	79	46,XY[20]	*TET2*, *RUNX1 SRSF*2	Hydroxyurea Hypomethylating agent 5 (HMA) Low cytarabine
2	87	Woman	AML	MDS (RAEB)	BM	68.20	12.96	20	46, XX[20]	n.d.	Erythropoietin Hypomethylating agent 5 (HMA)
3	69	Men	AML	CMML	PB	28	5.32	38	46, XY, del(20) (qq11q13) [44]/46, sl, t(8; 21) (q22; q22) [5]/46, sd11, der (22) t(8; 22) (q21-q22; p12) [11]	*ASXL1*, *SRSF2*	Hypomethylating agent 5 (HMA) AML-type chemotherapy
4	65	Men	AML	CMML	PB	17.9	5.7	25	46, XY [20]	*RUNX1*, *ASXL1*, *ZRSR2*, *CSF3R*	AML-type chemotherapy Hypomethylating agent 5 (HMA)
5	76	Men	AML	None	BM	36.40	6.55	59	46, XY[20]	n.d.	Hydroxyurea
6	68	Men	AML	MPN *Polycythemia vera*	PB	43	5.59	64	46, XY[22]	*JAK2*	Hypomethylating agent 5 (HMA)

WHO: World health organization; AML: Acute myeloid leukemia; CMML: Chronic myelomonocytic leukemia; MDS: Myelodysplastic syndromes; RAEB: Refractory anemia with excess of blasts; MPN: Myeloproliferative neoplasms; WBC: Whole blood cells; PB: peripheral blood; BM: Bone marrow; del: deletion; sl: stemline; sd: sideline ; der: derived; t: translocation; n.d.: not determined.

Supplementary Table 2 Characteristics of the T-ALL patients providing the PDX cells used in this study.

**Patients**	**Age at sampling time (years)**	**Sex**	**WHO diagnosis**	**Karyotypes**	**Additional gene mutations**	**Multi-agent received treatments**
PDX#1	16	M	T-ALL	46,XY,t(11;14)(p13;q11),add(13)(p11)[8]/46,XY[19] .nuc ish(ABL1,BCR)x2[200],(TCRADx2)(5'TCRAD sep 3'TCRADx1)[117/200]	$IL7R^{\mu}$<br>$NOTCH1^{\mu}$	None
PDX#2	17	M	T-ALL	46,XY,t(11;14)(p13;q11),add(13)(p11)[8]/46,XY[19] .nuc ish(ABL1,BCR)x2[200],(TCRADx2)(5'TCRAD sep 3'TCRADx1)[117/200]	$IL7R^{\mu}$<br>$NOTCH1^{\mu}$<br>$PTPRG^{del}$<br>$HOXA11^{del}$<br>$CDKN2A^{del}$<br>$CDKN2B^{del}$<br>$TCRD^{del}$<br>$NF1^{gain}$<br>HLF^{gain}	Daratumomab<br>Venetoclax<br>Dexamethasone<br>Nelarabine<br>Vincristine<br>Cytarabine<br>Etoposide<br>GCSF
PDX#3	6	F	T-ALL	46,XX,t(11;14)(p13;q11)	SIL-TAL1<br>$CDKN2A^{del}$<br>$RB1^{del}$<br>$NOTCH1^{\mu}$	None

WHO: World health organization; T-ALL: T-Cell acute lymphoblastic leukemia; PDX: Patient derived Xenograft; M: Male; F: Female; t: translocation; del: deletion; add: addition; µ: mutation; nuc ish: nuclear in situ hybridization
