# Supplementary material for: CDKN1A is a target for phagocytosis-mediated cellular immunotherapy in acute leukemia
Source: Nat Commun. 2022 Nov 8;13:6739. doi: 10.1038/s41467-022-34548-3 (PMC9643439; doi:10.1038/s41467-022-34548-3)
Supplement: Supplementary file 3 — Description of Additional Supplementary Files [file 41467_2022_34548_MOESM3_ESM.pdf]

Description of Additional Supplementary Files

Supplementary Data 1. List of upregulated and downregulated genes in Phago+ MDMs as compared to Phago- MDMs (in Fig. 1j).
