# Supplementary material for: CDKN1A is a target for phagocytosis-mediated cellular immunotherapy in acute leukemia
Source: Nat Commun. 2022 Nov 8;13:6739. doi: 10.1038/s41467-022-34548-3 (PMC9643439; doi:10.1038/s41467-022-34548-3)
Supplement: Supplementary file 4 — Supplementary Data 1 [file 41467_2022_34548_MOESM4_ESM.pdf]

Supplementary Data 1. List of upregulated and downregulated genes in Phago^{+} MDMs as compared to Phago^{-} MDMs (in Fig. 1j).

Accession number	Gene Name	Donor-1 Log2FC-1	Donor-1 Log2FC-2	Donor-2 Log2FC-1	Donor-2 Log2FC-2	Donor-3 Log2FC-1	Donor-3 Log2FC-2	Mean Log2FC	Standard deviation	References
NM_001243962	HLA-DQB1	-0.581085461	-0.51949771	-0.543775531	-0.402907544	-0.900165827	-0.901591057	-0.641503862	0.209554892	
NM_001031804	MAF	-0.748971298	-0.757355501	-0.195699637	-0.154685012	-0.751059946	-0.651663441	-0.543239139	0.288053769	1
NM_006895	HNMT	-1.153512746	-1.312527881	-0.182960439	-0.14665632	-0.728895472	-0.878054827	-0.733767947	0.485814816	2
NM_002003	FCN1	-0.441026188	-0.416885858	-0.230801825	-0.318938384	-0.208387703	-0.141453228	-0.292915531	0.119938729	3
NM_001946	DUSP6	-0.051145628	-0.215729214	-0.270050542	-0.170089954	-0.548420144	-0.465961842	-0.286899554	0.187098811	4
NM_001001547	CD36	-0.495976027	-0.52489668	-0.493696546	-0.487506267	-0.550731852	-0.520427366	-0.51220579	0.024203199	2
NM_033554	HLA-DPA1	-0.968770986	-0.99228559	-0.138121342	0.005934016	-1.482841483	-1.369283679	-0.824228177	0.62288117	
NM_002122	HLA-DQA1	-0.852073536	-0.773154495	-0.635247778	-0.765620669	-0.972414139	-0.997311877	-0.832637082	0.1371314	
NM_153811	SLC38A6	-0.496021681	-0.470885344	-0.447700281	-0.370343873	-0.541073635	-0.658669451	-0.497449044	0.09719409	2
NM_004244	CD163	-1.496105316	-1.650303955	-0.580943975	-0.544766047	-1.300798552	-1.113780799	-1.114449774	0.464046325	5
NM_001150	ANPEP	-1.07333231	-1.156206103	-0.371499456	-0.59527098	-0.536939154	-0.441411901	-0.695776651	0.334583748	6
NM_005623	CCL8	-0.763508565	-0.788545586	-0.906444943	-0.667630277	-0.564061212	-0.508529916	-0.69978675	0.148782288	
NM_018092	NETO2	-1.340147798	-1.292177359	-0.316902202	-0.332758573	-1.185540585	-1.036854232	-0.917396792	0.47069006	
NM_004235	KLF4	-0.518527327	-0.430745078	-0.28226564	-0.08419777	-0.137030314	-0.170882209	-0.270608056	0.173078469	7
NM_138711	PPARG	-0.606230518	-0.627521049	-0.687632794	-0.690680448	-0.388590406	-0.436133474	-0.572798115	0.129464495	8
NM_002124	HLA-DRB1	-0.631505148	-0.612412789	-0.371562307	-0.344473955	-0.986503653	-0.936111262	-0.647094852	0.271172379	
NM_019111	HLA-DRA	-0.394418097	-0.417530794	-0.302401288	-0.32702838	-0.619917993	-0.524004868	-0.43088357	0.12100183	
NM_002982	CCL2	-4.034458272	-4.122824587	-0.330785733	-0.355001943	-0.277198599	-0.285232608	-1.567583624	1.945470015	
NM_005755	EBI3	-0.49050625	-0.628082925	-0.164509195	-0.211666767	-0.881780624	-0.913493255	-0.548339836	0.320904403	
NM_001562	IL18	-0.296245809	-0.343382573	-0.1780507	-0.167052357	-0.45377753	-0.444585523	-0.313849082	0.124775453	9
NM_002984	CCL4	-1.262445528	-1.259288658	-0.583295898	-0.585440701	-0.080994996	-0.074718382	-0.641030694	0.530880867	
NM_002852	PTX3	-2.690391917	-2.724744491	-1.292646305	-1.309115891	-1.453141202	-1.402132423	-1.812028705	0.696285375	
NM_003596	TPST1	-0.774306308	-0.674300928	-0.123652374	-0.092013878	-0.164570028	-0.046455513	-0.312549838	0.322832918	1
NM_000129	F13A1	-1.142032748	-1.037272543	-0.038868858	-0.131280679	-1.497818827	-1.312429134	-0.859950465	0.620928529	
NM_002731	PRKACB	-1.011072338	-1.032191533	-0.223821059	-0.384355538	-0.521610881	-0.425795189	-0.599807756	0.34066848	10
NM_001482	GATM	-0.340103432	-0.446443832	-0.601126918	-0.621274302	-1.441684205	-1.437187712	-0.814636733	0.494870001	11
NM_000677	ADORA3	-1.57763162	-1.591203995	-0.356265318	-0.370834656	-1.649875119	-1.92649118	-1.245383648	0.694675261	1
NM_020362	PITHD1	-0.656463418	-0.608586573	-0.132963386	-0.198773236	-0.464364381	-0.441486462	-0.417106243	0.212221943	5
NM_013308	GPR171	-1.062439912	-0.971339566	-2.701184225	-2.470683152	-0.683938626	-0.820587156	-1.45169544	0.891018294	12
NM_002371	MAL	-0.843058848	-0.704240041	-3.041125348	-2.943123983	-0.8856428	-1.049665515	-1.577809422	1.101488978	
NM_001178126	IGLL5	-1.226899044	-1.011679171	-2.538817678	-2.613049755	-1.501288483	-1.488248219	-1.729997058	0.680266297	
NM_006159	NELL2	-0.675280278	-0.507305661	-2.334335332	-2.488018985	-0.726561502	-0.647794551	-1.229882718	0.919193428	
NM_002258	KLRB1	-0.554364779	-0.740102335	-2.00808683	-2.136960424	-1.071601332	-1.250285814	-1.293566919	0.652033148	
NM_014767	SPOCK2	-1.704071577	-1.857451462	-2.636960299	-2.701015041	-1.668554471	-1.717989094	-2.047673657	0.485959639	
NM_198196	CD96	-0.696125208	-0.593621022	-2.231885364	-2.09452307	-0.842749897	-0.825060622	-1.213994197	0.742101926	
ENST00000390237	IGKC	-1.610846846	-1.291948829	-2.789431718	-2.773346202	-2.07316802	-2.035264304	-2.095667653	0.60427258	
NM_001768	CD8A	-0.662776678	-0.89732213	-2.535116732	-2.411008205	-0.956368469	-1.062611052	-1.420867211	0.826418592	
NM_002341	LTB	-1.613354977	-1.225439473	-2.85302617	-2.94015058	-1.372357878	-1.403359095	-1.901281362	0.78133449	
NM_014207	CD5	-0.794298307	-0.722912376	-2.087569779	-2.106745623	-0.671205058	-0.678643314	-1.176895743	0.714200006	
NM_002104	GZMK	-0.444578248	-0.150299334	-2.282560308	-2.427657263	-0.529490154	-0.621743696	-1.076054834	1.004330829	
NM_014722	FAM65B	-0.620388577	-0.219090372	-2.008572585	-1.802580879	-0.268834554	-0.481008858	-0.900079304	0.794899899	
ENST00000390547	IGHA1	-0.374010217	-0.390645917	-2.162315619	-2.118474601	-1.39912612	-1.428199573	-1.312128674	0.790347938	
NM_148965	TNFRSF25	-1.440527616	-1.451045121	-3.083101823	-3.019569571	-1.30307197	-1.350649733	-1.941327639	0.861823909	
NM_006725	CD6	-1.19371842	-0.949347873	-2.047800435	-2.040130221	-0.679723787	-0.536437995	-1.241193122	0.661554374	
NM_001767	CD2	-2.793477034	-2.657363371	-4.380364013	-4.515877283	-2.078919972	-2.167563424	-3.098927516	1.081291773	
NM_017933	PID1	-1.334882199	-1.123752236	-0.182140465	-0.249230214	-2.330043267	-2.701564186	-1.320268761	1.040087832	
ENST00000390551	IGHG3	-0.295267099	-0.367642431	-0.710481179	-0.691586762	-0.341380192	-0.382887578	-0.464874207	0.185426285	
NM_152866	MS4A1	-0.669085636	-0.593621022	-0.965302399	-0.812678376	-0.749724315	-0.562576721	-0.725498078	0.150207002	
NM_003121	SPIB	-0.43060032	-0.330818667	-1.079752434	-0.555633815	-0.849450814	-0.777775329	-0.670671896	0.281832671	
NM_006235	POU2AF1	-0.846333813	-1.024642717	-1.504356826	-1.609182871	-1.184297532	-1.137936986	-1.217791791	0.289168721	
NM_152296	ATP1A3	1.065830065	1.153916169	1.079159224	0.93499379	0.380491665	0.226792188	0.80686385	0.399085765	
NM_004633	IL1R2	1.6867575	1.887059922	0.378575927	0.203046934	0.955640799	0.860995048	0.995346022	0.678376502	
NM_001172	ARG2	1.277183548	1.287324072	0.312664826	0.366667991	0.355630197	0.512124012	0.685265774	0.467293172	13
NM_004615	TSPAN7	0.677530199	0.833303	2.512598751	2.533954705	0.896725707	0.912759954	1.394478719	0.878332776	6
NM_002309	LIF	0.92385174	0.855900108	0.134783983	0.067470431	0.486896914	0.616873895	0.514296179	0.357598844	
NM_001025366	VEGFA	1.052609135	1.081569247	0.614795091	0.587387906	1.000890123	0.861532163	0.866463944	0.219196574	14
NM_000073	CD3G	0.683562372	0.722470193	0.395664755	0.563994874	0.2211598	0.155881262	0.457122209	0.23803691	
NM_000732	CD3D	1.939773492	2.114086946	1.432574455	1.507153427	1.167837383	1.255093409	1.569419852	0.378549184	
NM_000576	IL1B	0.120703651	0.117081911	0.352773716	0.294523198	1.290071109	1.278331349	0.575580822	0.556798987	15
NM_001008540	CXCR4	1.375972405	1.343364601	0.88041326	0.853668985	0.871708008	0.900460289	1.037597925	0.250141408	16
NM_015714	G0S2	0.981400318	0.960626553	1.040424186	1.01521941	2.245997479	2.028016295	1.37861404	0.592114454	
NM_021181	SLAMF7	0.697913337	0.910389113	0.064891268	0.08679108	0.329917011	0.244746172	0.389107997	0.342914175	17
NM_003486	SLC7A5	1.727712248	1.676075005	0.473277137	0.510149746	0.586725239	0.546154357	0.920015622	0.607028774	18
NM_004417	DUSP1	0.835203553	0.858259885	0.242787469	0.223760348	0.989660702	0.972497069	0.687028171	0.35674138	
NM_175839	SMOX	0.46750539	0.455103638	0.303217854	0.349663856	0.779522713	0.734016178	0.514838272	0.197975365	
NM_002192	INHBA	0.640279166	0.582721651	0.412412774	0.449761872	1.775233475	1.73246749	0.932146071	0.642092155	19
NM_003811	TNFSF9	1.264445984	1.08025231	0.512080969	0.481186666	1.092475687	1.002212273	0.905442315	0.328181072	
NM_001100812	CXCL16	1.503012272	1.468096755	0.212266686	0.235775919	0.629875745	0.648671242	0.78294977	0.575231637	15
NM_181054	HIF1A	0.948406259	0.839172103	0.399685798	0.395663352	1.28177456	1.283694902	0.858066162	0.39828027	20
NM_000963	PTGS2	0.581174412	0.509767842	0.104279728	0.098204146	0.161319789	0.107354948	0.260350144	0.223159691	13
NM_005375	MYB	3.499775743	3.389197836	4.530634632	4.841587902	2.152346479	2.039161628	3.408784037	1.164013717	5
NM_001661	ARL4D	2.435439484	2.476020048	0.434521353	0.538697448	1.310952908	1.239860346	1.405915264	0.88739048	
NM_001002915	IGFL2	2.709616395	2.671378259	0.217106968	0.284296147	1.155564423	1.290222678	1.388030812	1.099678956	
NM_182908	DHRS2	3.416933701	3.30810239	3.799972307	3.714671415	1.347452045	1.439846691	2.837829758	1.133725689	
NM_002728	PRG2	4.187722242	4.262844823	0.825942813	0.824745112	1.239202917	1.358372817	2.116471787	1.647745928	
NM_012250	RRAS2	2.422034083	2.348244032	0.73511902	0.962415028	0.73511902	0.962415028	1.360891035	0.800204723	21
NM_016269	LEF1	2.515079235	2.518435867	1.335993192	1.283038146	1.329125504	1.310715492	1.715397906	0.621001984	21 22

NM_020689	SLC24A3	2.509869054	2.487945778	0.24417411	0.29596393	0.855669424	0.686652881	1.180045863	1.047391562	23
NM_016459	MZB1	2.583394848	2.596940739	0.723107334	0.847418516	0.723107334	0.847418516	1.386897881	0.933715209	24
NM_014220	TM4SF1	3.684623887	3.664781802	1.28603437	1.322213046	1.28603437	1.322213046	2.094316753	1.224284799	
NM_002648	PIM1	2.426849072	2.423825868	0.429656285	0.366686471	1.503533027	1.466063122	1.436102308	0.907656914	
NM_001145033	AG2	3.301542123	3.32959608	1.297876972	1.280865528	1.297876972	1.280865528	1.964770534	1.046389336	
NR_026597	DIRC3	2.407209551	2.404685824	0.272469241	0.05800101	0.792124093	0.832259623	1.127791557	1.033770692	
NM_001172292	NIPAL4	3.697098425	3.724208584	0.386233055	0.357622115	2.441272989	2.528298357	2.189122254	1.510887475	
ENST00000380464	PLIN2	2.870003562	2.72541771	0.871565548	0.813998109	1.432938739	1.492002491	1.700987693	0.895128106	
NM_033120	NKD2	2.411773556	2.215007278	2.978825795	2.738906968	1.21440627	1.288940654	2.141310087	0.737945891	
NM_002404	MFAP4	2.736404754	2.75575098	1.847561408	1.806745917	0.977980575	1.007566721	1.855335059	0.784591106	
NM_021158	TRIB3	3.216638594	3.232921566	0.211681968	0.189167337	0.153481855	0.188647295	1.198756436	1.569470086	25
NM_139314	ANGPTL4	3.330031124	3.44457626	1.140097981	0.91885867	1.183578945	1.19680129	1.868990712	1.180912556	
NM_000597	IGFBP2	1.396431888	1.434434584	2.865212024	2.81262923	1.226250159	1.166577261	1.816922524	0.798159749	
NM_001853	COL9A3	1.076008673	1.19733003	3.400763795	3.217354319	1.457647379	1.231997201	1.930183566	1.076741237	
NM_001005464	HIST2H3A	0.233668323	0.164024855	2.432292745	2.451638157	1.347452045	1.439846691	1.344820469	1.004551206	
NM_018667	SMPD3	1.538149607	1.624892199	3.043850324	2.911168449	0.859509374	1.013238869	1.83180147	0.935792179	26
NM_018492	PBK	0.354317436	0.235393346	2.57989656	2.694358302	0.529973589	0.422286034	1.136037545	1.167226926	
NM_004867	ITM2A	2.267706361	2.28824222	2.570112004	2.646682524	0.152224439	0.197676711	1.687107376	1.180949555	
NR_033916	CCDC26	1.467317138	1.522604404	3.902704634	3.934413137	1.358916834	1.179927602	2.227647291	1.315025728	
NM_001004317	LIN28B	1.271591361	1.399339878	3.158333171	3.24915891	1.364422982	1.546020693	1.998144499	0.938460457	27
NM_002612	PDK4	1.884871379	1.962521516	1.552354138	1.426121895	2.388744452	2.28023544	1.915808136	0.382493573	28

Fold changes (FC) are shown.

References of Supplementary Data 1

1. Kang, K. *et al.* Interferon-gamma Represses M2 Gene Expression in Human Macrophages by Disassembling Enhancers Bound by the Transcription Factor MAF. *Immunity* **47**, 235-250 e234, doi:10.1016/j.immuni.2017.07.017 (2017).

2. Martinez, F. O., Gordon, S., Locati, M. & Mantovani, A. Transcriptional profiling of the human monocyte-to-macrophage differentiation and polarization: new molecules and patterns of gene expression. *J Immunol* **177**, 7303-7311, doi:10.4049/jimmunol.177.10.7303 (2006).

3. Honore, C. *et al.* The innate pattern recognition molecule Ficolin-1 is secreted by monocytes/macrophages and is circulating in human plasma. *Mol Immunol* **45**, 2782-2789, doi:10.1016/j.molimm.2008.02.005 (2008).

4. Carson, W. F. t. *et al.* Enhancement of macrophage inflammatory responses by CCL2 is correlated with increased miR-9 expression and downregulation of the ERK1/2 phosphatase Dusp6. *Cell Immunol* **314**, 63-72, doi:10.1016/j.cellimm.2017.02.005 (2017).

5. Cuevas, V. D. *et al.* MAFB Determines Human Macrophage Anti-Inflammatory Polarization: Relevance for the Pathogenic Mechanisms Operating in Multicentric Carpotarsal Osteolysis. *J Immunol* **198**, 2070-2081, doi:10.4049/jimmunol.1601667 (2017).

6. Gundra, U. M. *et al.* Alternatively activated macrophages derived from monocytes and tissue macrophages are phenotypically and functionally distinct. *Blood* **123**, e110-122, doi:10.1182/blood-2013-08-520619 (2014).

7. Liao, X. *et al.* Kruppel-like factor 4 regulates macrophage polarization. *J Clin Invest* **121**, 2736-2749, doi:10.1172/JCI45444 (2011).

8. Desterke, C., Turhan, A. G., Bennaceur-Griscelli, A. & Griscelli, F. PPARgamma Cistrome Repression during Activation of Lung Monocyte-Macrophages in Severe COVID-19. *iScience* **23**, 101611, doi:10.1016/j.isci.2020.101611 (2020).

9. Yasuda, K., Nakanishi, K. & Tsutsui, H. Interleukin-18 in Health and Disease. *Int J Mol Sci* **20**, doi:10.3390/ijms20030649 (2019).

10. Na, Y. R. *et al.* Protein Kinase A Catalytic Subunit Is a Molecular Switch that Promotes the Pro-tumoral Function of Macrophages. *Cell Rep* **31**, 107643, doi:10.1016/j.celrep.2020.107643 (2020).

11. Jha, A. K. *et al.* Network integration of parallel metabolic and transcriptional data reveals metabolic modules that regulate macrophage polarization. *Immunity* **42**, 419-430, doi:10.1016/j.immuni.2015.02.005 (2015).

12. Schridde, A. *et al.* Tissue-specific differentiation of colonic macrophages requires TGFbeta receptor-mediated signaling. *Mucosal Immunol* **10**, 1387-1399, doi:10.1038/mi.2016.142 (2017).

13. Ashley, J. W. *et al.* Polarization of Macrophages toward M2 Phenotype Is Favored by Reduction in iPLA2beta (Group VIA Phospholipase A2). *J Biol Chem* **291**, 23268-23281, doi:10.1074/jbc.M116.754945 (2016).

14. Granata, F. *et al.* Production of vascular endothelial growth factors from human lung macrophages induced by group IIA and group X secreted phospholipases A2. *J Immunol* **184**, 5232-5241, doi:10.4049/jimmunol.0902501 (2010).

15. Orecchioni, M., Ghosheh, Y., Pramod, A. B. & Ley, K. Macrophage Polarization: Different Gene Signatures in M1(LPS+) vs. Classically and M2(LPS-) vs. Alternatively Activated Macrophages. *Front Immunol* **10**, 1084, doi:10.3389/fimmu.2019.01084 (2019).

16. Tian, X. *et al.* CXCR4 knockdown prevents inflammatory cytokine expression in macrophages by suppressing activation of MAPK and NF-kappaB signaling pathways. *Cell Biosci* **9**, 55, doi:10.1186/s13578-019-0315-x (2019).

17. Simmons, D. P. *et al.* SLAMF7 engagement superactivates macrophages in acute and chronic inflammation. *Sci Immunol* **7**, eabf2846, doi:10.1126/sciimmunol.abf2846 (2022).

18. Yoon, B. R., Oh, Y. J., Kang, S. W., Lee, E. B. & Lee, W. W. Role of SLC7A5 in Metabolic Reprogramming of Human Monocyte/Macrophage Immune Responses. *Front Immunol* **9**, 53, doi:10.3389/fimmu.2018.00053 (2018).

19. Sierra-Filardi, E. *et al.* Activin A skews macrophage polarization by promoting a proinflammatory phenotype and inhibiting the acquisition of anti-inflammatory macrophage markers. *Blood* **117**, 5092-5101, doi:10.1182/blood-2010-09-306993 (2011).

20. Wang, T. *et al.* HIF1alpha-Induced Glycolysis Metabolism Is Essential to the Activation of Inflammatory Macrophages. *Mediators Inflamm* **2017**, 9029327, doi:10.1155/2017/9029327 (2017).

21. Borrell-Pages, M., Romero, J. C., Juan-Babot, O. & Badimon, L. Wnt pathway activation, cell migration, and lipid uptake is regulated by low-density lipoprotein receptor-related protein 5 in human macrophages. *Eur Heart J* **32**, 2841-2850, doi:10.1093/eurheartj/ehr062 (2011).

22. Xue, J. *et al.* Transcriptome-based network analysis reveals a spectrum model of human macrophage activation. *Immunity* **40**, 274-288, doi:10.1016/j.immuni.2014.01.006 (2014).

23. Villa-Bellosta, R., Hamczyk, M. R. & Andres, V. Novel phosphate-activated macrophages prevent ectopic calcification by increasing extracellular ATP and pyrophosphate. *PLoS One* **12**, e0174998, doi:10.1371/journal.pone.0174998 (2017).

24. Zhang, H., Chen, X. & Sairam, M. R. Novel hormone-regulated genes in visceral adipose tissue: cloning and identification of proinflammatory cytokine-like mouse and human MEDA-7: implications for obesity, insulin resistance and the metabolic syndrome. *Diabetologia* **54**, 2368-2380, doi:10.1007/s00125-011-2212-7 (2011).

25. Riera-Borrull, M. *et al.* Palmitate Conditions Macrophages for Enhanced Responses toward Inflammatory Stimuli via JNK Activation. *J Immunol* **199**, 3858-3869, doi:10.4049/jimmunol.1700845 (2017).

26. Al-Rashed, F. *et al.* Neutral sphingomyelinase 2 regulates inflammatory responses in monocytes/macrophages induced by TNF-alpha. *Sci Rep* **10**, 16802, doi:10.1038/s41598-020-73912-5 (2020).

27. Jaiswal, A., Maurya, M., Maurya, P. & Barthwal, M. K. Lin28B Regulates Angiotensin II-Mediated Let-7c/miR-99a MicroRNA Formation Consequently Affecting Macrophage Polarization and Allergic Inflammation. *Inflammation* **43**, 1846-1861, doi:10.1007/s10753-020-01258-1 (2020).

28. Min, B. K. *et al.* Pyruvate Dehydrogenase Kinase Is a Metabolic Checkpoint for Polarization of Macrophages to the M1 Phenotype. *Front Immunol* **10**, 944, doi:10.3389/fimmu.2019.00944 (2019).
