# Supplementary material for: CDKN1A is a target for phagocytosis-mediated cellular immunotherapy in acute leukemia
Source: Nat Commun. 2022 Nov 8;13:6739. doi: 10.1038/s41467-022-34548-3 (PMC9643439; doi:10.1038/s41467-022-34548-3)
Supplement: Supplementary file 5 — Reporting Summary [file 41467_2022_34548_MOESM5_ESM.pdf]

nature portfolio

Corresponding author(s): Awatef ALLOUCH
Jean-Luc PERFETTINI

Last updated by author(s): Aug 25, 2022

Reporting Summary

Nature Portfolio wishes to improve the reproducibility of the work that we publish. This form provides structure for consistency and transparency in reporting. For further information on Nature Portfolio policies, see our Editorial Policies and the Editorial Policy Checklist.

Statistics

For all statistical analyses, confirm that the following items are present in the figure legend, table legend, main text, or Methods section.

n/a	Confirmed	
☐	☒	The exact sample size (*n*) for each experimental group/condition, given as a discrete number and unit of measurement
☐	☒	A statement on whether measurements were taken from distinct samples or whether the same sample was measured repeatedly
☐	☒	The statistical test(s) used AND whether they are one- or two-sided<br>*Only common tests should be described solely by name; describe more complex techniques in the Methods section.*
☐	☒	A description of all covariates tested
☐	☒	A description of any assumptions or corrections, such as tests of normality and adjustment for multiple comparisons
☐	☒	A full description of the statistical parameters including central tendency (e.g. means) or other basic estimates (e.g. regression coefficient) AND variation (e.g. standard deviation) or associated estimates of uncertainty (e.g. confidence intervals)
☐	☒	For null hypothesis testing, the test statistic (e.g. *F*, *t*, *r*) with confidence intervals, effect sizes, degrees of freedom and *P* value noted<br>*Give P values as exact values whenever suitable.*
☒	☐	For Bayesian analysis, information on the choice of priors and Markov chain Monte Carlo settings
☒	☐	For hierarchical and complex designs, identification of the appropriate level for tests and full reporting of outcomes
☒	☐	Estimates of effect sizes (e.g. Cohen's *d*, Pearson's *r*), indicating how they were calculated

Our web collection on statistics for biologists contains articles on many of the points above.

Software and code

Policy information about availability of computer code

Data collection	Confocal images LASX (Leica), FACSDiva version 8 (BD), Microarray images Extraction software version (10.7.3.1) (Agilent Technologies), GeneSys software version v1.3.9.0 (Genesys), qPCR CFX Maestro Version 4.0.2325.0418. (Biorad).
Data analysis	LASX (Leica), Imaris 5.7 software (Bitplane AG), GraphPad Prism 8.0 (GraphPad Software,Inc.), FACSDiva version 8 (BD), Microarray raw intensities limma package for R v2.13.0 (Agilent Technologies), Gene tools from Syngene version 4.02.03 (Syngene), qPCR CFX Maestro Version 4.0.2325.0418. (Biorad), Volocity software 6.2 (Quorum Technologies).

For manuscripts utilizing custom algorithms or software that are central to the research but not yet described in published literature, software must be made available to editors and reviewers. We strongly encourage code deposition in a community repository (e.g. GitHub). See the Nature Portfolio guidelines for submitting code & software for further information.

Data

Policy information about availability of data

All manuscripts must include a data availability statement. This statement should provide the following information, where applicable:

- Accession codes, unique identifiers, or web links for publicly available datasets
- A description of any restrictions on data availability
- For clinical datasets or third party data, please ensure that the statement adheres to our policy

The data generated in this study are provided in the Article, Supplementary Information or Source Data file. The microarray gene expression data of the modulated genes in phagocytic macrophages with respect to non-phagocytic macrophages are provided in Fig. 1j, Supplementary Data 1 and Source Data file. The microarray gene expression large dataset is available in ArrayExpress repository under the access code E-MTAB-12280 [https://www.ebi.ac.uk/biostudies/studies/E-MTAB-12280]. The SIRPA 2 promoter sequence (EP026655) is available in the Eukaryotic Promoter Database (EPD) (https://epd.epfl.ch//index.php). Source data

are provided with this paper.

Field-specific reporting

Please select the one below that is the best fit for your research. If you are not sure, read the appropriate sections before making your selection.

- [x] Life sciences
- [ ] Behavioural & social sciences
- [ ] Ecological, evolutionary & environmental sciences

For a reference copy of the document with all sections, see nature.com/documents/nr-reporting-summary-flat.pdf

Life sciences study design

All studies must disclose on these points even when the disclosure is negative.

Sample size	No sample size calculation was performed. The sample size was chosen based on the biological variability between human donors, mice and the statistical significance of the obtained results. Experiments with primary macrophages were performed from at least n=3 independent healthy donors. Mice experiments were performed from at least n=3 NSG mice/group. Experiments on cell lines were performed from at least n=3 independent experiments.
Data exclusions	No data were excluded from the analysis.
Replication	Experiments were performed with primary monocytes/macrophages from independent healthy donors. Macrophage phagocytosis experiments were performed with different target cell lines and primary leukemic cells from different patients. The efficacy of the adoptive transfer of p21TD-Mos into hT-ALL leukemic NSG mice was assayed using prophylactic and curative approaches, involving the engraftement of T-ALL cell line or Patient-Derived-Xenografts (PDXs). The reproducibility of the experiments was verified by the significance of the statistical tests. All attempts at replication were successful.
Randomization	NSG Mice were randomized for equivalent body weights before adoptive transfer of Co.TD-Mos or p21TD-Mos. PDX NSG mice were randomized for equivalent percentages of bone marrow PDX cell engraftments before adoptive transfer of Co.TD-Mos or p21TD-Mos. For all in vitro studies, samples were allocated randomly.
Blinding	The data collection of flow cytometry experiments, confocal microscopy image acquisitions and quantifications of mice organs were blinded. For the in vitro phagocytosis experiments and in vivo experiments, investigators were not blinded to group allocation and data collection because prior validations of p21 and/or SIRPα overexpression and/or knockdowns in macrophages were necessary to conduct the experiments. The data reported in these experiments are not subjective.

Reporting for specific materials, systems and methods

We require information from authors about some types of materials, experimental systems and methods used in many studies. Here, indicate whether each material, system or method listed is relevant to your study. If you are not sure if a list item applies to your research, read the appropriate section before selecting a response.

Materials & experimental systems

n/a	Involved in the study	
[ ]	[x]	Antibodies
[ ]	[x]	Eukaryotic cell lines
[x]	[ ]	Palaeontology and archaeology
[ ]	[x]	Animals and other organisms
[ ]	[x]	Human research participants
[x]	[ ]	Clinical data
[x]	[ ]	Dual use research of concern

Methods

n/a	Involved in the study	
[x]	[ ]	ChIP-seq
[ ]	[x]	Flow cytometry
[x]	[ ]	MRI-based neuroimaging

Antibodies

Antibodies used

Anti-p21 Waf1/Cip1 (12D1) (Cell signalling, Cat#2947, Lot#11, diluted 1:1000, 20 µg /One reaction of Chip-qPCR assay)
Anti-Myosin Light Chain 2 (MLC2) (Cell signalling, Cat#3672, Lot#6, diluted 1:500)
Anti-Phospho (Ser19) Myosin light chain 2 (MLCS19*) (Cell signalling, Cat#3671, Lot#6, diluted 1:500)
Anti-αTubulin (DM1A) (Cell signalling, Cat#3873, Lot#3, diluted 1:1000)
Anti-α/β-Tubulin (Cell signalling, Cat#2148, Lot#4, diluted 1:1000)

Anti-IL8 (Abcam, Cat#ab106350, Lot#GR37368-40, diluted 1:500)
Anti-IRF5 (Abcam, Cat#ab21689, Lot#GR277112-1, diluted 1:1000)
Anti-ILβ (Abcam, Cat#ab2105, Lot#GR3208882-18, diluted 1:1000)
Anti-iNOS (Abcam, Cat#ab3523, Lot#GR3431006-2, diluted 1:500)

Anti-hIFNγ antibody (RD systems, Cat#MAB285, Clone: 25718, Lot#KW1718081, 1 μg/ml for in vitro Transwell cell cocultures, 100 μg/mouse for in vivo experiments)
Mouse IgG2A control isotype (RD systems, Cat# MAB003, 1 μg/ml for Transwell cell cocultures, 100 μg/mouse for in vivo experiments)
Anti-IL6 antibody (RD systems, Cat#AB-206-NA, Lot#AQ15 111, diluted 1:500)

Anti-hSIRPα antibody (Invitrogen, Cat#PA1-30537, Lot#VG3025430C, diluted 1:2000)
Anti-hCD45 antibody (Invitrogen, Cat#MA5-37809, Clone: JE03-05, Lot#WG3326934B, diluted 1:50)
FITC anti-hCD47 (Invitrogen, Cat#11-0479-42, Clone: B6H12, Lot#2296858, diluted 1:100)
APC anti-hSIRPα (Invitrogen, Cat#17-1729-42, Clone: 15-414, Lot#2138101, diluted 1:100)
APC anti-mF4/80 (Invitrogen, Cat#17-4801-82, Clone: BM8, Lot#2123749, diluted 1:100)
Alexa Fluor 700 anti-mMHC II (I-A/I-E) (Invitrogen, Cat#56-5321-82, Clone: M5/114.15.2, Lot#22101930, diluted 1:100)
PE-Cyanine7 anti-hCD71 (Transferrin receptor) (Invitrogen, Cat#25-0719-42, Clone: OKT9, Lot#2299248, diluted 1:100)

Anti-GAPDH antibody (EMD Millipore, Cat#MAB374, Lot#3725986, diluted 1:1000)

Anti-p53 (DO-1) antibody (Santa Cruz Biotechnology, Cat#sc-126, Lot#F113, diluted 1:1000)
Anti-LAMP2 (H4B4) antibody (Santa Cruz Biotechnology, Cat#sc-18822, Lot#C2613, diluted 1:100)

InVivoMAb anti-human CD47 antibody (Bio Cell, Cat#BE0019-1, Clone: B6.H12, Lot#741820J2, 7 μg/ml for in vitro cocultures, 100 μg/mouse for in vivo experiments)
Mouse IgG1 control isotype (Cat#BE0083) (Bio Cell, Cat#BE0083, Clone: MOPC-21, Lot#722920J3, 7 μg/ml for in vitro cocultures, 100 μg/mouse for in vivo experiments)

PE anti-hCD14 (eBioscience, Cat#12-0149-42, Clone: 61D3, Lot #4272024, diluted 1:100)

APC/Cy7 anti-hCD11b (BioLegend, Cat#301342, Clone: ICRF44, Lot#B262652, diluted 1:100)
PE anti-hCD34 (BioLegend, Cat#343606, Clone: 561, Lot#B265379, diluted 1:100)
PE/Dazzle 594 anti-hCD7 (BioLegend, Cat#343120, Clone: CD7-6B7, Lot#B288583, diluted 1:100)

Alexa Fluor 647 anti-hCD163 (BD Pharmingen, Cat#562669, Clone: GHI/61, Lot#42826446, diluted 1:100)
PE anti-hCD71 (BD Pharmingen, Cat#555537, Lot#4241832, diluted 1:100)
FITC anti-hCD206 (BD Pharmingen, Cat#551135, Lot#3011756, diluted 1:100)
PE Cy7 anti-hCD56 (BD Pharmingen, Cat#560916, Clone: B159, Lot#3070545, diluted 1:100)
FITC anti-hCD3 (BD Pharmingen, Cat#555339, Lot#3035946, diluted 1:100)
APC anti-hCD20 (BD Pharmingen, Cat#559776, Lot#3060541, diluted 1:100)
PE anti-hCD47 (BD Pharmingen, Cat#558046, Lot#3161991, diluted 1:100)
V450 anti-mCD45 (BD Horizon, Cat#560501, Clone: 30-F11, Lot#5141660, diluted 1:200)
APC anti-hCD45 (BD Pharmingen, Cat#555485, Lot#4184772, diluted 1:50)
BUV395 anti-hCD45 (BD Horizon, Cat#563792, Clone: HI30, diluted 1:200)
Alexa-Fluor 647 anti-hCD68 (BD Pharmingen, Cat#562111, Clone: Y1/82A, Lot#7201918, diluted 1:500)

Validation

All primary antibodies were used based on manufacturere's instructions, validations and in line with the relevant cited references on their websites. The primary antibodies were used for: western blot (anti-p21 Waf1/Cip1 (12D1) (Cell signalling, Cat#2947), anti-Myosin Light Chain 2 (MLC2) (Cell signalling, Cat#3672), anti-Phospho (Ser19) Myosin light chain 2 (MLC2S19*) (Cell signalling, Cat#3671), anti-αTubulin (DM1A) (Cell signalling, Cat#3873), anti-α/β-Tubulin (Cell signalling, Cat#2148), anti-IL8 (Abcam, Cat#ab106350), anti-IRF5 (Abcam, Cat#ab21689), anti-ILβ (Abcam, Cat#ab2105), anti-iNOS (Abcam, Cat#ab3523), anti-IL6 antibody (RD systems, Cat#AB-206-NA), anti-hSIRPα antibody (Invitrogen, Cat#PA1-30537), anti-GAPDH antibody (EMD Millipore, Cat#MAB374), anti-p53 (DO-1) antibody (Santa Cruz Biotechnology, Cat#sc-126)); immunofluorescence (anti-LAMP2 (H4B4) antibody (Santa Cruz Biotechnology, Cat#sc-18822), anti-hCD45 antibody (Invitrogen, Cat#MA5-37809, Clone: JE03-05), Alexa-Fluor 647 anti-hCD68 (BD Pharmingen, Cat#562111, Clone: Y1/82A), anti-iNOS (Abcam, Cat#ab3523)), flow cytometry (FITC anti-hCD47 (Invitrogen, Cat#11-0479-42, Clone: B6H12), APC anti-hSIRPα (Invitrogen, Cat#17-1729-42, Clone: 15-414), APC anti-mF4/80 (Invitrogen, Cat#17-4801-82, Clone: BM8), Alexa Fluor 700 anti-mMHC II (I-A/I-E) (Invitrogen, Cat#56-5321-82, Clone: M5/114.15.2), PE-Cyanine7 anti-hCD71 (Transferrin receptor) (Invitrogen, Cat#25-0719-42, Clone: OKT9), PE anti-hCD14 (eBioscience, Cat#12-0149-42, Clone: 61D3), APC/Cy7 anti-hCD11b (BioLegend, Cat#301342, Clone: ICRF44), PE anti-hCD34 (BioLegend, Cat#343606, Clone: 561), PE/Dazzle 594 anti-hCD7 (BioLegend, Cat#343120, Clone: CD7-6B7), Alexa Fluor 647 anti-hCD163 (BD Pharmingen, Cat#562669, Clone: GHI/61), PE anti-hCD71 (BD Pharmingen, Cat#555537), FITC anti-hCD206 (BD Pharmingen, Cat#551135), PE-Cy7 anti-hCD56 (BD Pharmingen, Cat#560916, Clone: B159), FITC anti-hCD3 (BD Pharmingen, Cat#555339), APC anti-hCD20 (BD Pharmingen, Cat#559776, Lot#3060541), PE anti-hCD47 (BD Pharmingen, Cat#558046), V450 anti-mCD45 (BD Horizon, Cat#560501, Clone: 30-F11), APC anti-hCD45 (BD Pharmingen, Cat#555485) and BUV395 anti-hCD45 (BD Horizon, Cat#563792, Clone: HI30); in vitro and in vivo blocking assays (anti-hIFNγ antibody (RD systems, Cat#MAB285, Clone: 25718) and inVivoMAb anti-human CD47 antibody (Bio Cell, Cat#BE0019-1, Clone: B6.H12)).

Eukaryotic cell lines

Policy information about cell lines

Cell line source(s)

Jurkat, MOLT4, CEM, HEL and K562, HEK293T cell lines were purchased from and validated by the American Type Culture Collection (ATCC).

Authentication	The cell lines were not authenticated.
Mycoplasma contamination	Routine mycoplasma tests were performed by MycoAlert mycoplasma detection kit (LONZA). All cell lines were negative for mycoplasma.
Commonly misidentified lines (See ICLAC register)	None of the cell lines used in this manuscript are listed in the ICLAC database of cross-contaminated or misidentified cell lines.

Animals and other organisms

Policy information about studies involving animals; ARRIVE guidelines recommended for reporting animal research

Laboratory animals	Cg-PrkdcscidIl2rgtm1Wjl/SzJ (NSG) mice were obtained from Charles Rivers laboratories. NSG mice (6 to 8 weeks old male and female) were maintained in Gustave Roussy Institute Animal facility (France) in specific pathogen-free (SPF) grade animal room at 20-22°C ambient temperature, humidity (45-60%) and 12h (7:00 a.m.-7:00 p.m.) light/dark cycle. Mouse studies were performed in accordance with protocols approved by the French Ethical Committee Comité d'Ethique en Expérimentation Animale N°026 (CEEA26) of the Ministère de l'éducation nationale, de l'enseignement supérieur et de la recherche (Project N°2012-022) and following recommendations for proper use and care during animal experimentation.
Wild animals	The study did not involve wild animals.
Field-collected samples	The study did not involve samples collected from the field.
Ethics oversight	Mice studies were performed in accordance with protocols approved by the French Ethical Committee CEEA26 and following recommendations of proper use and care of animal experimentation.

Note that full information on the approval of the study protocol must also be provided in the manuscript.

Human research participants

Policy information about studies involving human research participants

Population characteristics	Monocyte-derived macrophages (MDMs) and primary blood lymphocytes (PBLs) used in this study are from buffy coats of healthy donors (aged 25-69 years old, 50% of men and 50% of women) from French blood bank (Etablissement Français du sang). The characteristics of Acute Myeloid Leukemia (AML) patients, from which were obtained CD34 cells used in this study, are described in Supplementary Table 1 (aged 65-87 years old, 83.33% of men and 16.67 % of women). The characteristics of T-cell Acute Lymphoblastic Leukemia (T-ALL) patients, from which were generated patient derived xenograft (PDX) cells used in this study, are described in Supplementary Table 3 (aged 6-17 years old, 66.66% of male 33.33% female).
Recruitment	Healthy donors are selected by French blood bank (EFS) according to the healthy immune-hematological characteristics and negativity to HIV, HCV, HBV, CMV and HTLV infections. No self selection bias of healthy donors is included in this study. AML patients were selected in the context of MYELOMONO2 study of Groupe Francophone des Myelodysplasies (France) according to the World Health Organization (WHO) diagnosis criteria of AML disease. No self selection bias of AML patients is included in this study. T ALL patients were selected as pediatric or young adult patients according to the WHO diagnosis criteria of T-ALL disease by the pediatric hematological departements from hospital Armand Trousseau and hospital Robert Debré (Paris , France). No self selection bias of T-ALL patients is included in this study.
Ethics oversight	Healthy donors, AML patients or T ALL patient parents or legal representatives gave informed consents in accordance with French law and the Declaration of Helsinki. Human samples were obtained from (i) healthy donors from the French blood bank, after approval of Etablissement Français du Sang (EFS) ethical committee (convention N° 14EFS003), (ii) AML patients after the approval of the CCTIRS (Comité Consultatif sur le Traitement de l'Information en matière de Recherche dans le domaine de la Santé) committee ethical approval authorization (CCTIR N°14-266) and the CNIL (Commission Nationale de l'Informatique et des Libertés) committee ethical approval authorization (CNIL N°914283) and (iii) T ALL patients after the approval of the ethical review board (IRB00003888) of Institut National de la Santé et de la Recherche Médicale (INSERM) (project N° 13-105-2) for experimentations with T ALL samples.

Note that full information on the approval of the study protocol must also be provided in the manuscript.

Flow Cytometry

Plots

Confirm that:

- [x] The axis labels state the marker and fluorochrome used (e.g. CD4-FITC).
- [x] The axis scales are clearly visible. Include numbers along axes only for bottom left plot of group (a 'group' is an analysis of identical markers).
- [x] All plots are contour plots with outliers or pseudocolor plots.
- [x] A numerical value for number of cells or percentage (with statistics) is provided.

Methodology

Sample preparation	Cell stainings were performed in DPBS containing 2% (for in vitro experiments) or 5% (for mouse models) HI FBS at 4°C for 2h incubation with 1:100 antibody dilution. For blood samples obtained from engrafted mice, red cells were lysed with ACK buffer before incubations with antibodies and/or FACS analysis for mCherry+ or CFSE+ cells. Mouse cell samples were filtered through 100 µm pore size membrane filters before FACS staining and analyses.
Instrument	Cell samples were analyzed by FACS using BD LSR2 or BD LSRFortessa (BD Biosciences) and sorted using BD FACSAria III (BD Biosciences).
Software	FACS DIVA version 8 (BD).
Cell population abundance	Purity of macrophages and cell lines sorted with BD FACSAria III (BD Biosciences) was of 98-99%.
Gating strategy	FACS gating was performed on positivity or negativity of cellular markers of interest following classical FCS/SSC and singlet live cell gatings.

☒ Tick this box to confirm that a figure exemplifying the gating strategy is provided in the Supplementary Information.
